# METTL3 dual regulation of the stability of LINC00662 and VEGFA RNAs promotes colorectal cancer angiogenesis

**DOI:** 10.1007/s12672-022-00557-3

**Published:** 2022-09-17

**Authors:** Guoying Zhang, Tianjun Wang, Zihui Huang, Yuanyuan Chen, Li Sun, Xia Xia, Fang He, Chenying Fan, Shukui Wang, Wanli Liu

**Affiliations:** 1 Department of General Clinical Research Center, Nanjing First Hospital, Nanjing Medical University, No. 68, Changle Road, Nanjing, 210006 Jiangsu China; 2 Department of Clinical Laboratory, Nanjing Integrated Traditional Chinese and Western Medicine Hospital, Nanjing University of Traditional Chinese Medicine, No. 179, Xiaolingwei Street, Nanjing, 210014 Jiangsu China; 3 Department of Obstetrics and Gynecology, The First Affiliated Hospital of Nanjing Medical University, Nanjing, Jiangsu China; 4 Department of Biochemistry and Molecular Biology, Nanjing Medical University, Nanjing, Jiangsu China; 5 Jiangsu Collaborative Innovation Center on Cancer Personalized Medicine, Nanjing Medical University, Nanjing, Jiangsu China

**Keywords:** Colorectal cancer angiogenesis, METTL3, LINC00662, VEGFA, *N*6-methyladenosine (m6A)

## Abstract

**Purpose:**

The angiogenesis is among the primary factors that affect tumor recurrence and distant organ metastasis in colorectal cancer (CRC). *N*6-methyladenosine (m6A) modification is one of the most common chemical modifications in eukaryotic mRNA, especially at the post-transcriptional level. Methyltransferase-like 3 (METTL3) promoting angiogenesis in a variety of tumors has been reported. However, the mechanism of how METTL3 dual-regulates the stability of long non-coding RNAs (lncRNAs) and vascular-related factor RNAs to affect angiogenesis in CRC is unclear.

**Methods:**

64 paired CRC and adjacent normal tissues were collected. In vitro, quantitative real-time polymerase chain reaction (qRT-PCR), immunohistochemistry (IHC), actinomycin assay, methylated RNA immunoprecipitation (MeRIP) experiment,3-(4,5)-dimethylthiahiazo(-z-y1)-3,5-di-phenytetrazoliumromide (MTT) and colony formation assay were performed. The functions were also studied in zebrafish model animals in vivo.

**Results:**

We found that the vascular endothelial growth factor A(VEGFA), METTL3 and LINC00662 RNAs were highly expressed in CRC, and that METTL3 was significantly positively correlated with LINC00662 and VEGFA. The protein expression levels of CD31, CD34, VEGFA, m6A and METTL3 were all significantly increased in the CRC tissues. The angiogenesis experiments both in vivo and in vitro found that METTL3 and LINC00662 promoted angiogenesis in CRC. The actinomycin assay indicated that METTL3 maintained the stability of LINC00662 and VEGFA RNAs. In addition, the MeRIP experiment confirmed that the LINC00662 and VEGFA RNAs had METTL3-enriched sites.

**Conclusion:**

These findings suggest that METTL3 and LINC00662 may both serve as diagnostic and prognostic predictive biomarkers for CRC and potential targets for anti-vascular therapy.

**Supplementary Information:**

The online version contains supplementary material available at 10.1007/s12672-022-00557-3.

## Background

Colorectal cancer (CRC) is the fourth most common cancer worldwide, and its incidence is on the rise [1]. Despite advances in the diagnosis and treatment of CRC over the past few decades, its mortality remains high, and this is primarily due to recurrence and distant organ metastases [2].

Recent studies have found that angiogenesis in the tumor microenvironment is critical to the recurrence and distant organ metastasis of CRC because blood is required to provide essential oxygen and nutrients during these processes [3–5]. Currently, anti-angiogenesis therapy has been one of the most commonly used methods for the treatment of tumor recurrence and drug-resistant distant organ metastasis. However, the 5-year survival rate of patients after standardized anti-vascular therapy has not significantly improved [6]. Studies regarding the mechanism of angiogenesis in CRC are mostly focused on the transcriptional level [7–9]. Therefore, there is an urgent need to explore new mechanisms of CRC angiogenesis, especially at the post-transcriptional level, so as to provide a new theoretical basis for anti-vascular therapy.

Epigenetic regulatory mechanisms, such as DNA methylation, histone methylation, and acetylation or *N*6-methyladenosine (m6A), are emerging research frontiers in tumor biology [10–13]. As the most abundant post-transcriptional modification, m6A has become an important regulator of mRNA, affecting various basic biological processes [14]. methyltransferase-like 3 (METTL3) is a key member of the m6A methyltransferase complex that has recently been reported to play an important role in influencing angiogenesis and promoting tumor progression [15–19]. However, the mechanism by which METTL3 promotes angiogenesis in CRC remains unexplored.

LncRNAs, as non-coding RNAs longer than 200 nt, are involved in a variety of biological processes. Recent studies have found that they can affect tumor angiogenesis and play an important role in promoting tumor recurrence and distant organ metastasis [4, 20, 21]. In this study, LINC00662, a lncRNA with a length of 2097 nt, was found to be located on the long arm of chromosome 19. In the TCGA (The Cancer Genome Atlas) CRC database, LINC00662 was highly expressed in CRC and was also significantly positively correlated with VEGFA.

In this study, we first demonstrated that METTL3, LINC00662, and vascular endothelial growth factor A(VEGFA) were significantly positively correlated not only in the TCGA CRC database, but also in CRC tissue specimens. It was also confirmed that both METTL3 and LINC00662 promoted angiogenesis in CRC. In addition, MeRIP experiments confirmed that METTL3 dually regulated the stability of the LINC00662 and VEGFA RNAs to maintain their expression, thereby promoting angiogenesis in CRC.

## Methods

### Clinical tissue specimens

Sixty-four CRC and 64 adjacent normal tissues were obtained during surgery from the Nanjing First Hospital, which is affiliated with the Nanjing Medical University. The operation to collect tissue samples from patients is from 2019 to 2021. The diagnosis of CRC and adjacent normal tissues was confirmed according to the pathological evidence. Tissues were snap-frozen in liquid nitrogen and stored at − 80 °C before detection was performed. This study was approved by the Ethics Committee of the Nanjing Medical University, and informed consent was obtained from all participants.

### Cell culture and transfection

Normal colon cells (FHC), human umbilical vein endothelial cells (HUVEC), and CRC cells (HCT116, HT29, SW480, and DLD1; Shanghai Cell Bank, Shanghai, China) were cultured in Dulbecco’s Modified Eagle Medium supplemented with 10% fetal bovine serum (FBS) and penicillin/streptomycin (100 U/ml) in an incubator with 5% CO_2_ at 37 °C (the HUVEC cells were cultured in endothelial cell medium (ECM)). All of the cell lines used in this study were mycoplasma-free. Lipofectamine 2000 (Invitrogen, Carlsbad, California, USA) was used for cell siRNA transfection, and the X-Tremegene HP DNA transfection reagent (Roche, Mannheim, Germany) was used for cell plasmid transfection. During transfection, 50–70% of cells in the six-well plate should be transfected. Six hours after the siRNA transfection, the cells required a medium change, but not for the plasmid transfection. RNA was collected 24 h after cell transfection, and protein was collected 48 h after cell transfection.

### Plasmid and interference sequence construction

The siRNA interference sequences of LINC00662, METTL3, and insulin-like growth factor 2 mRNA binding protein 1 (IGF2BP1) are listed in Supplementary Table 1. The full lengths of the human METTL3 (NM_019852.5), VEGFA (NM_001025366.3), IGF2BP1 (NM_001160423.2), and LINC00662 (NR_027301.1) were also cloned into the pcDNA3.1(+) vector (Invitrogen # V80020). The primers are listed in Sect. 2.4.

### DNA, RNA extraction and qRT-PCR

The plasmids METTL3, LINC00662, and VEGFA were extracted from *E. coli* using the Endo free Plasmids Mini Kit II (50) Kit (OMEGA, Norcross, GA, USA). The total RNA was extracted from the CRC cell lines or fresh frozen samples using the Trizol reagent (Invitrogen, Carlsbad, California, USA), and the cDNA was reversed transcribed using the PrimeScript RT Reagent Kit (Takara, Shiga, Japan) according to the manufacturer’s protocol and stored at − 20 °C. Quantitative real-time PCR was performed in triplicate in the Step One Plus TM real-time PCR Instrument (Applied Biosystems by Thermo Fisher Scientific, Singapore). GAPDH served as the internal reference gene. The primers used in this paper are listed in Table 1.

**Table 1:** The primers used in this study

Gene	Sequence (5ʹ-3ʹ)
GAPDH	Forward primer TGGTATGAGAGCTGGGGAATG Reverse primer CCTCCCCACCTTGAAAGGAA
LINC00662	Forward primer ACGCTGCTGCCACTGTAATAA Reverse primer GTCCGCCTTTCACAGAACTGA
IGF2BP1	Forward primer GCGGCCAGTTCTTGGTCAA Reverse primer TTGGGCACCGAATGTTCAATC
VEGFA	Forward primer CGCTCGGTGCTGGAATTTGAT Reverse primer CCGTCGGCCCGATTCAAGT
METTL3	Forward primer TTGTCTCCAACCTTCCGTAGT Reverse primer CCAGATCAGAGAGGTGGTGTAG

### Angiogenesis assay in vitro

The pre-cooled 96-well plate was prepared in advance and placed on ice during the experiment. The normal concentration of the Corning gel thawed in advance was evenly separated in each well of the 96-well plate, and 50 μl was added to each well. The transfected cell supernatant was mixed with the HUVEC cells (3 × 10^4^) and dropped into the coagulated matrix glue. These were cultured in the incubator for 6–8 h to observe angiogenesis. Photos were taken (4×) under an Olympus microscope IX53 (Olympus, Center Valley, PA, USA). Image Pro was used to calculate the number of angiogenesis. Each experiment was analyzed in triplicate.

### Cell counting MTT assay and colony formation assay

After the transfection of the HCT116 and HT29 cells was completed, these cells were seeded on 96-well plates at a concentration of 3 × 104 cells and cultured for 0, 24, 48 and 72 h. Subsequently, 20 μl of the cell counting MTT was added to each well. After incubation at 37 °C for 4 h, the liquid was discarded and dimethyl sulfoxide (DMSO) was added. The absorbance value was then detected at 490 nm. After the transfection of the HCT116 and HT29 cells was completed, 3000–5000 cells were planted in each well of the six-well plate and cultured for 14 days. After methanol fixation, crystal violet staining was performed, and the number of clone cells was counted using Image J after photos were taken (4×) under an Olympus microscope IX53 (Olympus, Center Valley, USA). The concentration of crystal violet used in this experiment is 5‰. Each experiment was analyzed in triplicate.

### Western blotting

Cells were collected and lysed, and the protein concentration was determined. 10% SDS-PAGE gel was added into the protein, and electrophoresis was performed first. After marker separation, 0.45 μm PVDF membrane was transferred between the gel after methanol activated membrane. The protein was separated and transferred to a polyvinylidene difluoride membrane followed by incubation with 5% milk at 20 ± 5 °C for 1 h. The membrane was incubated at 4 °C overnight with the following antibodies: METTL3 (1:1000, AB195352, Abcam, Cambridge, MA, USA), VEGFA (1:1000, AB185238, Abcam, Cambridge, MA, USA), and GAPDH (1:1000, Beijing Zhongshan Golden Bridge Biotechnology Co. Ltd., Beijing, China). The secondary antibody was then added. The second antibody was sheep anti-rat and sheep anti-rabbit, and the dilution ratio was 1:5000 and the membrane was incubated at room temperature for 1 h. The protein expression was observed using a chemiluminescence gel imaging system (Tanon 5200, Shanghai, China).

### Immunohistochemistry

Tissue samples from the normal group and the CRC group were fixed and then cut into 4 μm sections for the IHC. In simple terms, the tissue samples were treated with ethylene diamine tetraacetic acid (EDTA) as an antigen extract and then treated with CD31 antibody (1:2000, AB76533, Abcam, Cambridge, MA, USA), CD34 antibody (1:400, AB81289, Abcam, Cambridge, MA, USA), VEGFA antibody (1:400, AB185238, Abcam, Cambridge, MA, USA), m6A antibody (1:200, AB151230, Abcam, Cambridge, MA,USA), METTL3 antibody (1:500, AB195352, Abcam, Cambridge, MA, USA), and IGF2BP1 antibody (1:4000, AB229700, Abcam, Cambridge, MA, USA), separately. These samples were then incubated overnight at 4 °C. Subsequently, the second antibody was incubated at 37 °C for 1 h. Finally, the samples were stained and imaged.

### m6A-qRT-PCR

The total RNA was extracted from the CRC cell lines using the Trizol reagent (Invitrogen, Carlsbad, California, USA). Approximately 100 μg of the total RNA was digested by DNase (Takara, Shiga, Japan) in a 150 μl reaction system at 37 °C for 20 min. After digestion, the total RNA was extracted again using the Trizol reagent, followed by RNA fragmentation using fragmentation reagents (Invitrogen, Carlsbad, California, USA) at 71 °C for 5 min. Then the termination buffer was added immediately. The fragmented RNA was extracted using the Trizol reagent and dissolved in 200 μl of diethylpyrocarbonate (DEPC) water. Approximately 160 μl of fragmented RNA was diluted with the MeRIP buffer (150 mM KCl, 25 mM Tris, 5 mM EDTA, 0.5% Triton X-100, 0.5 mM DTT, protease inhibitor (1:100) (Invitrogen, Carlsbad, California, USA), and RNAase inhibitor (1:1000) (ABclonal, Wuhan, China)) and divided into two tubes that were incubated with the anti-m6A antibody (ABclonal, Wuhan, China) or the control IgG antibody with protein A/G conjugated magnetic beads (MCE, Monmouth Junction, NJ, USA) in 900 μl of the RNA binding protein immunoprecipitation (RIP) lysis buffer at 4 °C for 4 h. In total, 20 ul of fragmented RNA was collected. The bound RNAs were immunoprecipitated with beads. The beads were washed with the RIP buffer four times and treated with 10 μl of 10% sodium dodecyl sulfate (SDS), 10 μl of proteinase K (Takara, Shiga, Japan), and 130 μl of the MeRIP buffer for 30 min at 55 °C. Then the treated liquid was transferred to new tubes. In each tube, 1 ml of the Trizol reagent and chloroform was added in turn. After centrifugation, the upper water phase was collected. A 1/10 volume of 3 M sodium acetate and an equal volume of isopropyl alcohol land glycogen with a final concentration of 100 ug/ml were added. After that, the samples were kept at − 80 °C overnight and then centrifuged at 12,000×*g* at 4 °C for 15 min. They were then washed twice with 75% ethanol. Finally, the precipitation was dissolved with an equal volume of DEPC water and analyzed using two-step quantitative RT-PCR (Takara, Shiga, Japan).

### RNA binding protein immunoprecipitation (RIP)

The CRC cells were washed twice with ice-cold PBS and lysed in 1 ml of the RIP Lysis buffer (150 mM KCl, 25 mM Tris, 5 mM EDTA, 0.5% Triton X-100, 0.5 mM DTT, protease inhibitor (1:100), and RNAase inhibitor (1:1000)) on ice for 30 min. The cell lysates were centrifuged at 12,000×*g* at 4 °C for 15 min. A total of 10% of the supernatant was collected, and the remaining supernatant was incubated with anti-METTL3 (Ab195352, Abcam, USA), anti-METTL16 (Ab252420, Abcam, USA), anti-ALKBH5 (ABE547, MERCK, USA), anti-FTO (ABE552, MERCK, USA), anti-YTHDC1 (Ab264375, Abcam, USA), anti-YTHDF1 (Ab220162, Abcam, USA), anti-YTHDF2 (Ab220163, Abcam, USA), anti-YTHDF3 (Ab220161, Abcam, USA), anti-IGF2BP1 (Ab184305, Abcam, USA), anti-IGF2BP2 (Ab128175, Abcam, USA), and anti-IGF2BP3 (Ab177477, Abcam, USA) antibody or the control IgG antibody with protein A/G conjugated magnetic beads (MCE, USA) in 900 μl of the RIP lysis buffer at 4 °C for 4 h. Bound RNAs were immunoprecipitated with beads. The beads were washed with RIP buffer four times and treated with 10 μl 10% SDS, 10 μl of proteinase K (Takara, Shiga Japan), and 130 μl of the MeRIP buffer for 30 min at 55 °C. RNA in the immunoprecipitation (IP) or input group was recovered with the Trizol reagent (Invitrogen, Carlsbad, California, USA) according to the manufacturer’s instruction and analyzed by quantitative RT-PCR. The enrichment ratio was calculated as a ratio of its amount in the IP to that in the input.

### Matrigel plug assay in nude mice

All animal care and procedures were conducted in accordance with the standards of the Administrative Regulations on Laboratory Animals approved by the State Council of the People’s Republic of China. The animal experiments were approved by the Ethics Committee of Experimental Animal Welfare of the Nanjing Medical University (IACUC-1705037). In this experiment, female BALB/c nude mice aged 8 weeks were purchased from the Viton lievera experiment and maintained in the condition without pathogens. The cells were resuspended in serum-free medium. Then, 0.2 ml of cell suspension was mixed with 0.2 ml of high-concentration matrix gel (BD Biosciences, San Jose, CA, USA), and the mixture was immediately injected subcutaneously into the back of nude mice. Mice were sacrificed 14 days after injection, and the matrix thrombus was removed. Next, photos were taken (4×) under an Olympus stereoscopic microscope MVX10 (Olympus, Center Valley, PA, USA). A total of 200 ml of FITC-conjugated lectin (1 mg/ml) was analyzed for capillary perfusion. The mice were injected intravenously 30 min before they were killed. The Drabkin reagent kit (Sigma-Aldrich) was used to analyze the hemoglobin according to the manufacturer’s instructions. The final hemoglobin concentration was measured at 540 nm and calculated according to the standard calibration curve. The expression levels of HE, CD34 and VEGFA were analyzed by immunohistochemistry.

### Zebrafish model animal

For the proliferation and migration studies, we chose the AB* strain zebrafish that were initially obtained from crosses between the AB strain (Zebrafish International Resource Center, ZIRC) and the local wild-type strain and subsequently inbred in the laboratory. A total of 48 h after cells were transfected in each well of a six-well plate, we used trypsin to digest and collect the cells and washed them twice with PBS. Then, we suspended the cells in a serum-free medium of 1 ml 1640 and counted the cells to make sure that the total number of cells was 1 × 10^6^. Next, we added 5 μl of the cell-labeling solution (Invitrogen) to per ml of the cell suspension and mixed the wells by gentle pipetting and incubated for 20 min at 37 °C. Furthermore, we cleaned the cells three times by 1640 serum-free medium and resuspended cells in 30 μl of DMEM culture medium and then injected. Four days later, photos were taken (4×) under an Olympus microscope IX53 (Olympus, Center Valley, USA). Each experiment was analyzed in triplicate.

### RNA stability assays

CRC cells were seeded in six-well plates overnight, and then treated with actinomycin D (5 μg/ml, HY-17559, MedChemExpress) at 0, 3, 6 and 9 h. The total RNA was then isolated by Trizol (Invitrogen, USA) and analyzed by qRT-PCR. The mRNA expression for each group at the indicated time was calculated and normalized by GAPDH. The mRNA half-lives time was estimated according to the linear regression analysis.

### Statistics and reproducibility

Each experiment was performed at least three biological replicates. Results are presented as the mean ± SD, comparisons were made using two-tailed Student’s t-test, two-tailed paired t-test, or two-tailed nonparametric Mann–Whitney U-test, and P < 0.05 indicates statistical significance (*P < 0.5; **P < 0.1; ***P < 0.01;****P < 0.001). Statistical analyses were performed using the GraphPad Prism 7 software. Quantization of the fluorescence area for proliferation and migration of zebrafish was performed by Image J software. The western blot results were quantified using Image J 1.53 software and normalized to the loading control or GAPDH.

## Results

### METTL3 was significantly positively correlated with LINC00662 and VEGFA

**Fig. 1:**
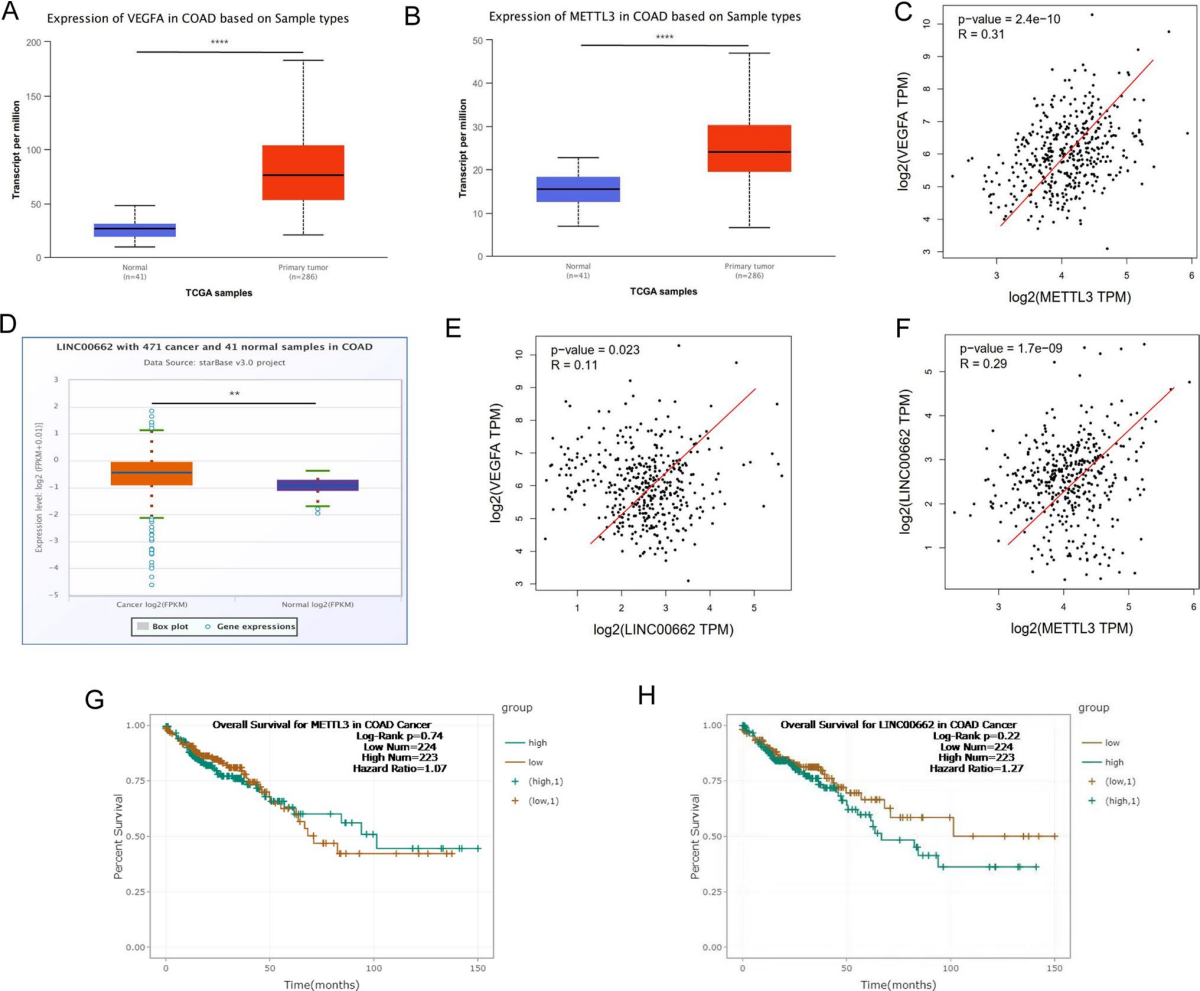
METTL3 was positively correlated with LINC00662 and VEGFA. **A** The expression of VEGFA mRNA in TCGA CRC database. **B** The expression of METTL3 mRNA in TCGA CRC database. **C** METTL3 mRNA was significantly positively correlated with VEGFA in TCGA CRC database. **D** LINC00662 mRNA was highly expressed in CRC in TCGA starBase database. **E** LINC00662 mRNA was significantly positively correlated with VEGFA in TCGA starBase database. **F** METTL3 mRNA was significantly positively correlated with LINC00662 in TCGA starBase database. **G** Survival curve analysis for METTL3 in CRC in TCGA CRC starBase database. **H** Survival curve analysis for LINC00662 in CRC in TCGA CRC starBase database

Based on the analysis of the TCGA CRC database (http://ualcan.path.uab.edu/analysis.html), we found that the mRNA levels of the vascular endothelial growth factor VEGFA and METTL3 in the CRC samples were high expressions (Fig. 1A, B), and METTL3 was significantly positively correlated with VEGFA (Fig. 1C). In addition, it was found that the expression level of the METTL3 protein was also increased in the CPTAC protein database (http://ualcan.path.uab.edu/analysis-prot.html) (Supplementary Fig. 1B). In addition, analysis in the TCGA CRC starBase database (https://starbase.sysu.edu.cn/) found that the LINC00662 mRNA was equally high expressed in the CRC (Fig. 1D) and significantly positively correlated with VEGFA (Fig. 1E). Further analysis found that METTL3 was also significantly positively correlated with LINC00662 (Fig. 1F). In addition, the RNA and protein interaction database (http://pridb.gdcb.iastate.edu/RPISeq/) forecasted the possibility of METTL3 interacting with LINC00662 was high (Supplementary Fig. 1A). Moreover, the survival curve analysis in the TCGA CRC starBase database showed that the higher expression levels of METTL3 and LINC00662, the shorter survival period of patients (Fig. 1G, H). The above results suggested that angiogenesis was significantly enriched in the CRC tissues when compared with para-cancer tissues. The METTL3 and LINC00662 expressions were increased in the CRC and both were significantly positively correlated with VEGFA, suggesting that they were related to angiogenesis in CRC.

### METTL3 positively correlated with LINC00662 and VEGFA

**Fig. 2:**
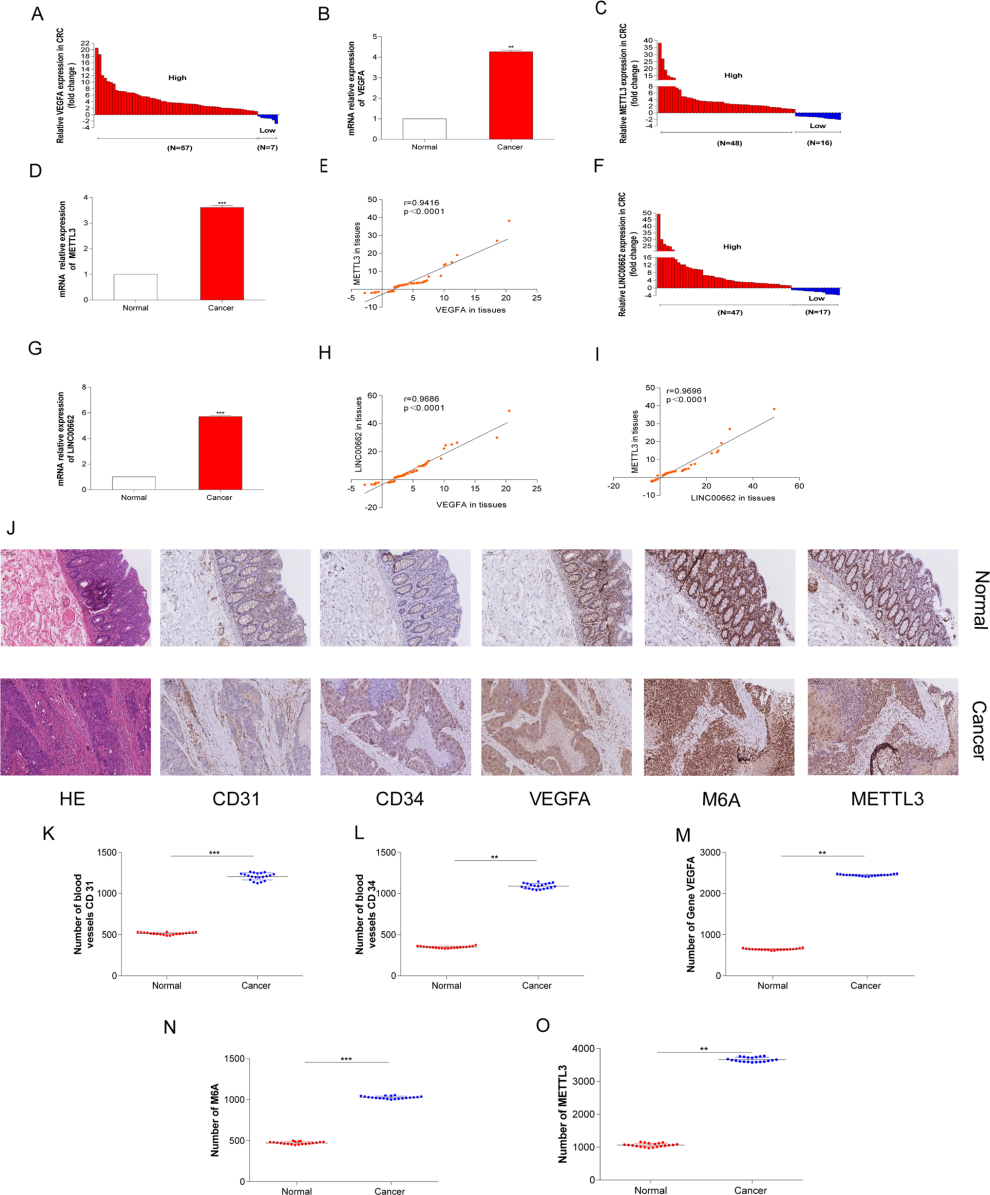
Based on tissue verification of CRC, METTL3 was positively correlated with LINC00662 and VEGFA. **A** The expression level of VEGFA mRNA in 64 pairs of CRC and para-cancer tissues. The expression levels of VEGFA were assessed, relative to expression of normal tissue. **B** The average expression level of VEGFA mRNA compared with para-cancer tissues. **C** The expression level of METTL3 mRNA in 64 pairs of CRC and para-cancer tissues. The expression levels of METTL3 were assessed, relative to expression of normal tissue. **D** The average expression level of METTL3 mRNA compared with para-cancer tissues. **E** Through statistical analysis, METTL3 mRNA was significantly positively correlated with VEGFA. **F** The expression level of LINC00662 mRNA in 64 pairs of CRC and para-cancer tissues. The expression levels of LINC00662 were assessed, relative to expression of normal tissue. **G** The average expression level of VEGFA mRNA compared with para-cancer tissues. **H** Through statistical analysis, LINC00662 mRNA was significantly positively correlated with VEGFA. **I** Through statistical analysis, METTL3 mRNA was significantly positively correlated with LINC00662. **J** Panoramic scans after HE staining for CD31, CD34, VEGFA, m6A and METTL3 in IHC experiments. **K** IHC analysis of CD31 in 20 pairs of CRC and para-cancer tissues. **L** IHC analysis of CD34 in 20 pairs of CRC and para-cancer tissues. **M** IHC analysis of VEGFA in 20 pairs of CRC and para-cancer tissues. **N** IHC analysis of m6A in 20 pairs of CRC and para-cancer tissues. **O** IHC analysis of METTL3 in 20 pairs of CRC and para-cancer tissues

In order to confirm that METTL3 and LINC00662 were related to the angiogenesis of CRC, 64 pairs of CRC and para-cancer tissues were collected in this study. The qRT-PCR result indicated that the levels of VEGFA, METTL3, and LINC00662 RNAs were highly expressed in CRC (Fig. 2A–B, F, G). METTL3 was significantly positively correlated with VEGFA and LINC00662, and LINC00662 was also significantly positively correlated with VEGFA (Fig. 2E, H, I), further confirming the role of the METTL3/LINC00662/VEGFA axis in promoting CRC angiogenesis. In addition, 20 pairs of CRC and adjacent tissues were selected for the IHC experiments. Compared with the adjacent tissues, the protein levels of CD31, CD34, and VEGFA related to blood vessels were significantly increased in CRC, and this indicated that angiogenesis was significantly increased in CRC also. Moreover, the levels of the m6A and m6A enzyme METTL3 were also obviously increased in CRC, suggesting that m6A may play a role in modifying RNAs in colorectal angiogenesis (Fig. 2J, O).

### METTL3 promoted colorectal cell angiogenesis

**Fig. 3:**
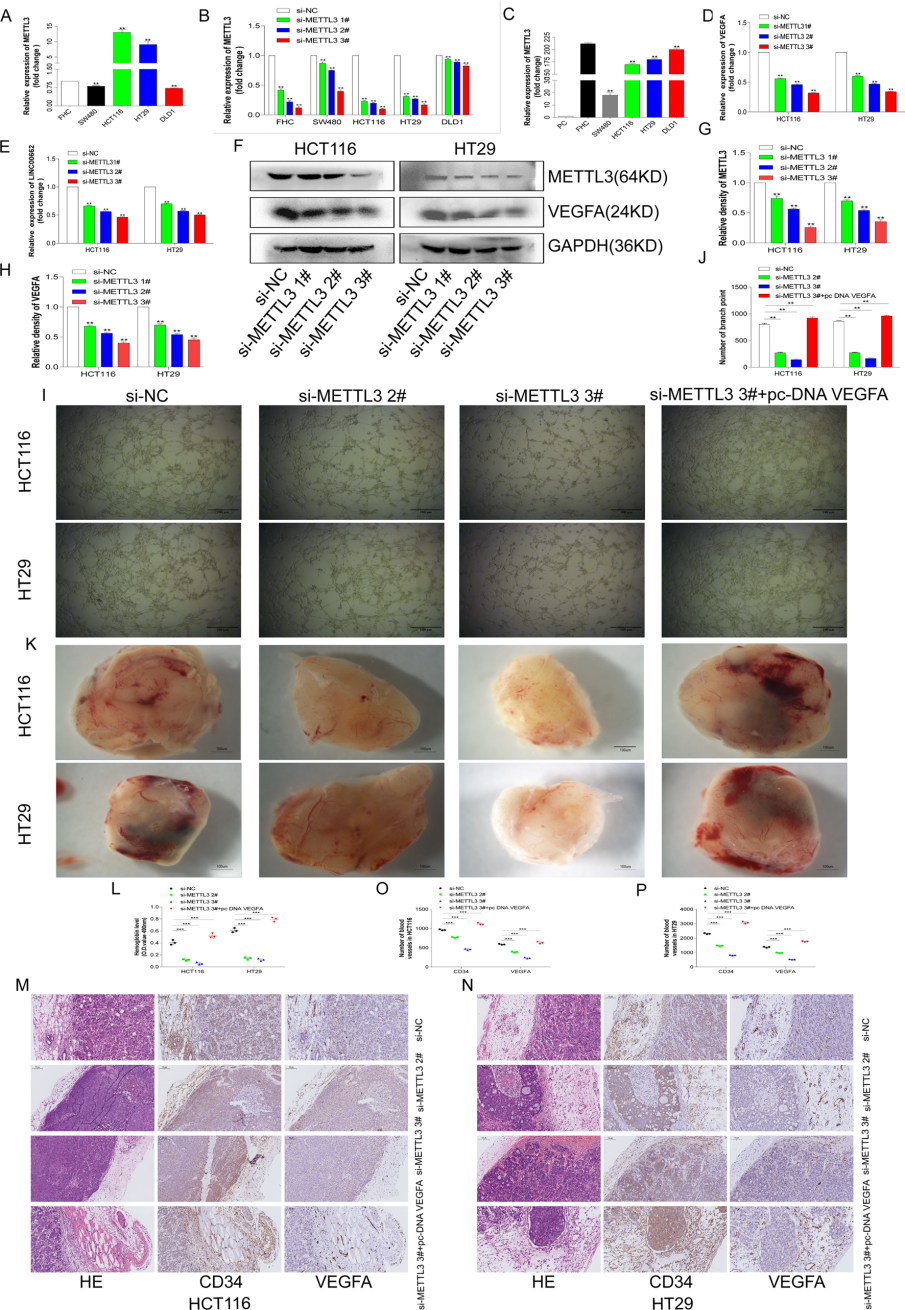
METTL3 promoted colorectal cell angiogenesis. **A** The expression levels of METTL3 in CRC cells SW480, HCT116, HT29 and DLD1, compared with colorectal normal cell FHC. **B** Interference efficiencies of METTL3 in FHC, SW480, HCT116, HT29 and DLD1. **C** Overexpression efficiencies of METTL3 in FHC, SW480, HCT116, HT29 and DLD1. **D** Relative expression of VEGFA mRNA after interfering with METTL3 in HCT116 and HT29 cells. **E** Relative expression of LINC00662 after interfering with METTL3 in HCT116 and HT29 cells. **F** METTL3 was interfered in HCT116 and HT29 cells, cell proteins were collected for western blot. METTL3 and VAGFA proteins were exposed. **G** Image J Quantified METTL3 protein level. **H** Image J quantified VEGFA protein level. **I** In vitro angiogenesis assay. **J** Image Pro quantified angiogenesis. **K** The Matrigel plug assay in nude mice. **L** Quantification of hemoglobin. **M** IHC staining and panoramic scanning for CD34 and VEGFA in HCT116 cell. **N** IHC staining and panoramic scanning for CD34 and VEGFA in HT 29 cell. **O** Image Pro quantifies of CD34 and VEGFA in HCT116 cell. **P** Image Pro quantifies of CD34 and VEGFA in HT29 cell

It has been confirmed above that METTL3 is related to angiogenesis in CRC. This study will further explore the effects of METTL3 on CRC cell angiogenesis at the cellular and animal levels. First, normal colorectal cells FHC, CRC cells SW480, HCT116, HT29 and DLD1 were recovered and cultured, and the cellular RNAs were collected. The results indicated that METTL3 was relatively highly expressed in CRC cells HCT116 and HT29 (Fig. 3A). Next, small interference sequences of METTL3 were designed to interfere with METTL3 in FHC, SW480, HCT116, HT29 and DLD1. The results showed that the interference efficiencies of METTL3 were relatively better in the HCT116 and HT29 cells (Fig. 3B). Finally, the METTL3 overexpression plasmid was constructed. METTL3 was overexpressed in the above cells, and it was found that METTL3 had the better overexpression efficiencies in FHC, HCT116, HT29 and DLD1 (Fig. 3C). Based on above results, the HCT116 and HT29 cells were selected for the next functional experiments.

First, in the HCT116 and HT29 cells, METTL3 was interfered and cell RNAs were collected. The qRT-PCR result showed that the levels of VEGFA and LINC00662 RNAs were significantly down-regulated (Fig. 3D, E). The proteins of the HCT116 and HT29 cells after interference with METTL3 were collected, and protein quantification showed that the METTL3 and VEGFA proteins were significantly down-regulated after METTL3 interference (Fig. 3F, H). These results suggested that METTL3 may regulate the expressions of LINC00662 and VEGFA in CRC. The supernatants of the HCT116 and HT29 cells transfected with si-NC, Si-METTL32#, si-METTL 3 3# and si-METTL3 3#+ pc DNA VEGFA were co-cultured and mixed with the HUVEC cells (3 × 10^4^). Cell angiogenesis was observed 8 h later on a 96-well plate containing a matrix gel (Fig. 3I). The quantitative results suggested that the angiogenesis of CRC cells was significantly decreased after METTL3 interference, but the overexpression of VEGFA significantly reversed the angiogenesis of CRC cells after METTL3 interference (Fig. 3J). In the in vivo animal studies of angiogenesis, the HCT116 and HT29 cells (1 × 10^7^) transfected with si-NC, si-METTL32#, si-METTL3 3# and si-METTL3 3#+ pc DNA VEGFA were mixed with a high concentration of matrix gel (BD Biosciences) on ice. Within 30 min, the mixed cell suspensions were injected into the subcutaneous area of the back of naked female mice. A total of 14 days later, the mice were sacrificed and a matrix glue plug was collected, as shown in Fig. 3K. Quantification of hemoglobin in the matrix glue plug showed that the angiogenesis level of the CRC cells decreased significantly after interference with METTL3. However, overexpression of VEGFA significantly reversed METTL3 interfering with angiogenesis (Fig. 3L). IHC staining for vascular related factors CD34 and VEGFA (Fig. 3M, N) was performed on the matrix rubber plug. The histochemical quantization results also suggested the above results (Fig. 3O, P).

### LINC00662 promotes colorectal cell angiogenesis

**Fig. 4:**
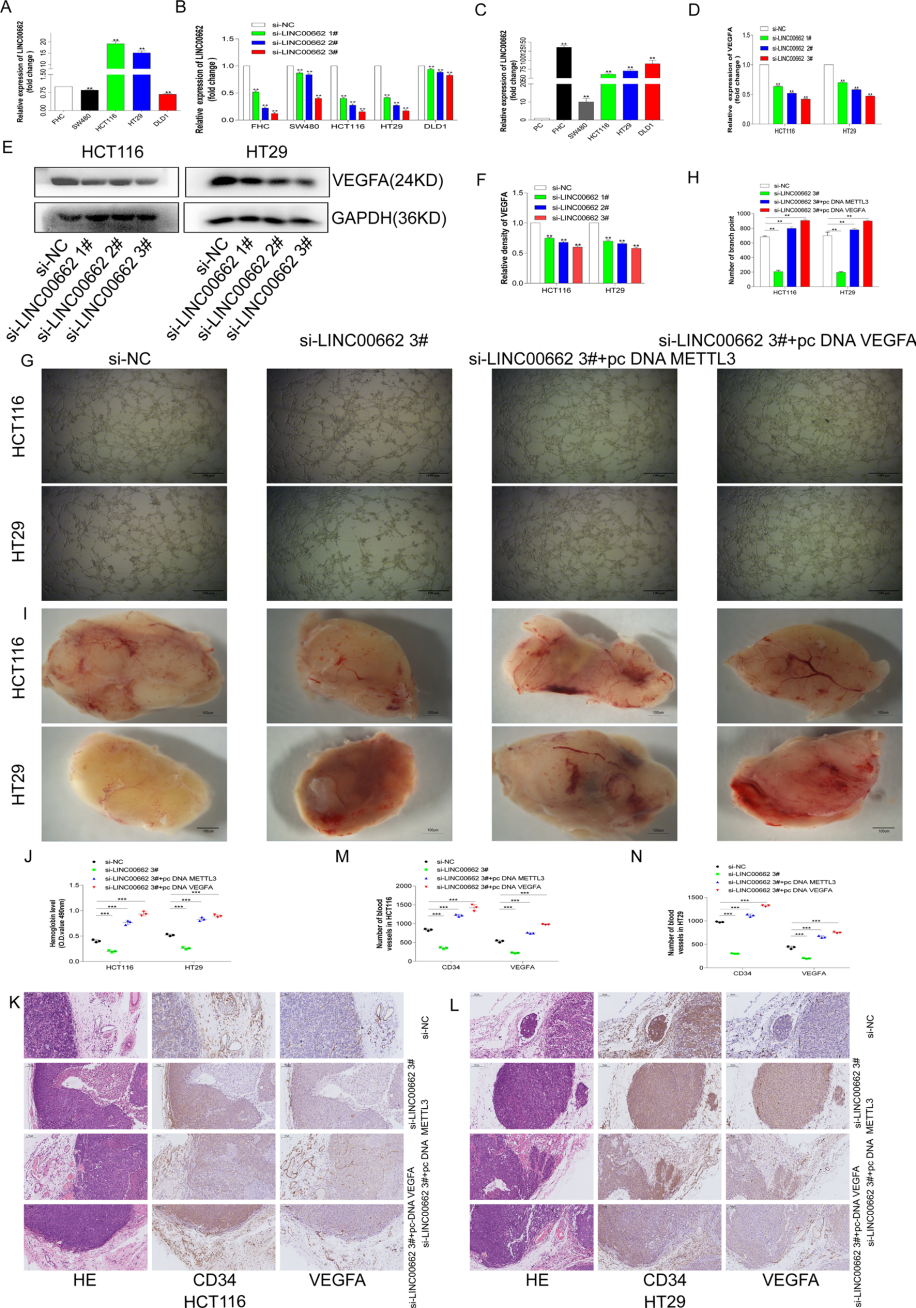
LINC00662 promotes colorectal cell angiogenesis. **A** The expression levels of LINC00662 in CRC cells SW480, HCT116, HT29 and DLD1, compared with colorectal normal cell FHC. **B** Interference efficiencies of LINC00662 in FHC, SW480, HCT116, HT29 and DLD1. **C** Overexpression efficiencies of LINC00662 in FHC, SW480, HCT116, HT29 and DLD1. **D** Relative expression of VEGFA mRNA after interfering with LINC00662 in HCT116 and HT29 cells. **E** Western blot of VEGFA after interfering with LINC00662 in HCT116 and HT29 cells. **F** Image J quantified VEGFA protein levels. **G** In vitro angiogenesis assay. **H** Image Pro quantification of angiogenesis. **I** The Matrigel plug assay in nude mice. **J** Quantification of hemoglobin. **K** IHC staining and panoramic scanning for CD34 and VEGFA in HCT116 cell. **L** IHC staining and panoramic scanning for CD34 and VEGFA in HT29 cell. **M** Image Pro quantifies of CD34 and VEGFA in HCT116 cell Matrigel plug. **N** Image Pro quantifies of CD34 and VEGFA in HT29 cell Matrigel plug

In the previous studies, it was confirmed that LINC00662 was also associated with angiogenesis in CRC. This study will further explore the effects of LINC00662 on the angiogenesis of CRC cells at the cellular and animal levels. First, normal colorectal cells FHC, CRC cells SW480, HCT116, HT29 and DLD1 were recovered and cultured, and the cellular RNAs were collected. Through qRT-PCR it was found that LINC00662 was also relatively highly expressed in the CRC cells HCT116 and HT29 (Fig. 4A). Next, we designed small interfering sequences of LINC00662 to interfere with LINC00662 in FHC, SW480, HCT116, HT29 and DLD1. It was found that the interference efficiencies of LINC00662 were relatively better in the HCT116 and HT29 cells (Fig. 4B). Finally, the LINC00662 overexpression plasmid was constructed, and LINC00662 was overexpressed in the above cells. It was found that the overexpression efficiencies of LINC00662 were relatively better in FHC, HCT116, HT29 and DLD1 (Fig. 4C). In summary, the HCT116 and HT29 cells were selected for the next functional experiments.

First, LINC00662 was disrupted in the HCT116 and HT29 cells, and the cellular RNAs were collected. The result of the qRT-PCR showed that the level of VEGFA mRNA was significantly down-regulated (Fig. 4D). Next, the HCT116 and HT29 cells after interference with LINC00662 were collected, and the western blot experiment was performed. It was found that the expression level of the VEGFA protein in the cells was significantly down-regulated after interference with LINC00662 (Fig. 4E, F). These results suggested that LINC00662 may regulate the expression of VEGFA in CRC. In the angiogenesis-related experiments in vitro and the supernatants of the HCT116 and HT29 cells transfected with si-NC, si-LINC00662 3#, si-LINC00662 3#+ pc DNA METTTL3 and si-LINC00662 3#+ pc DNA VEGFA were co-cultured and mixed with the HUVEC cells (3 × 10^4^) and then plated in a 96-well plate containing Matrigel. After 8 h, the angiogenesis was observed (Fig. 4G). The results suggested that the angiogenesis abilities of the CRC cells were significantly decreased after interference with LINC00662, but overexpression of METTL3 and VEGFA could significantly reverse the cellular angiogenesis after interfering with LINC00662 (Fig. 4H).

For in vivo animal studies of angiogenesis, the HCT116 and HT29 cells transfected with si-NC, si-LINC00662 3#, si-LINC00662 3#+ pc DNA METTTL3 and si-LINC00662 3#+ pc DNA VEGFA (1 × 10^7^) were mixed on ice with a high concentration of matrix gel (BD Biosciences, San Jose, CA, USA). Next, the mixed cell suspension was injected subcutaneously into the back of nude female mice within 30 min. After 14 days, the mice were sacrificed and the Matrigel plugs were collected as shown in Fig. 4I. The hemoglobin in the Matrigel plugs was quantified. After interfering with LINC00662, the angiogenesis level of the CRC cells was significantly decreased, but the overexpression of METTL3 and VEGFA could significantly reverse the angiogenesis after interfering with LINC00662 (Fig. 4J). Finally, the Matrigel plugs were stained with IHC for vascular-related factors CD34 and VEGFA (Fig. 4K, L). The histochemical quantification results also confirmed the above results (Fig. 4M, N).

### METTL3 dual regulated the LINC00662 and VEGFA RNA stability

**Fig. 5:**
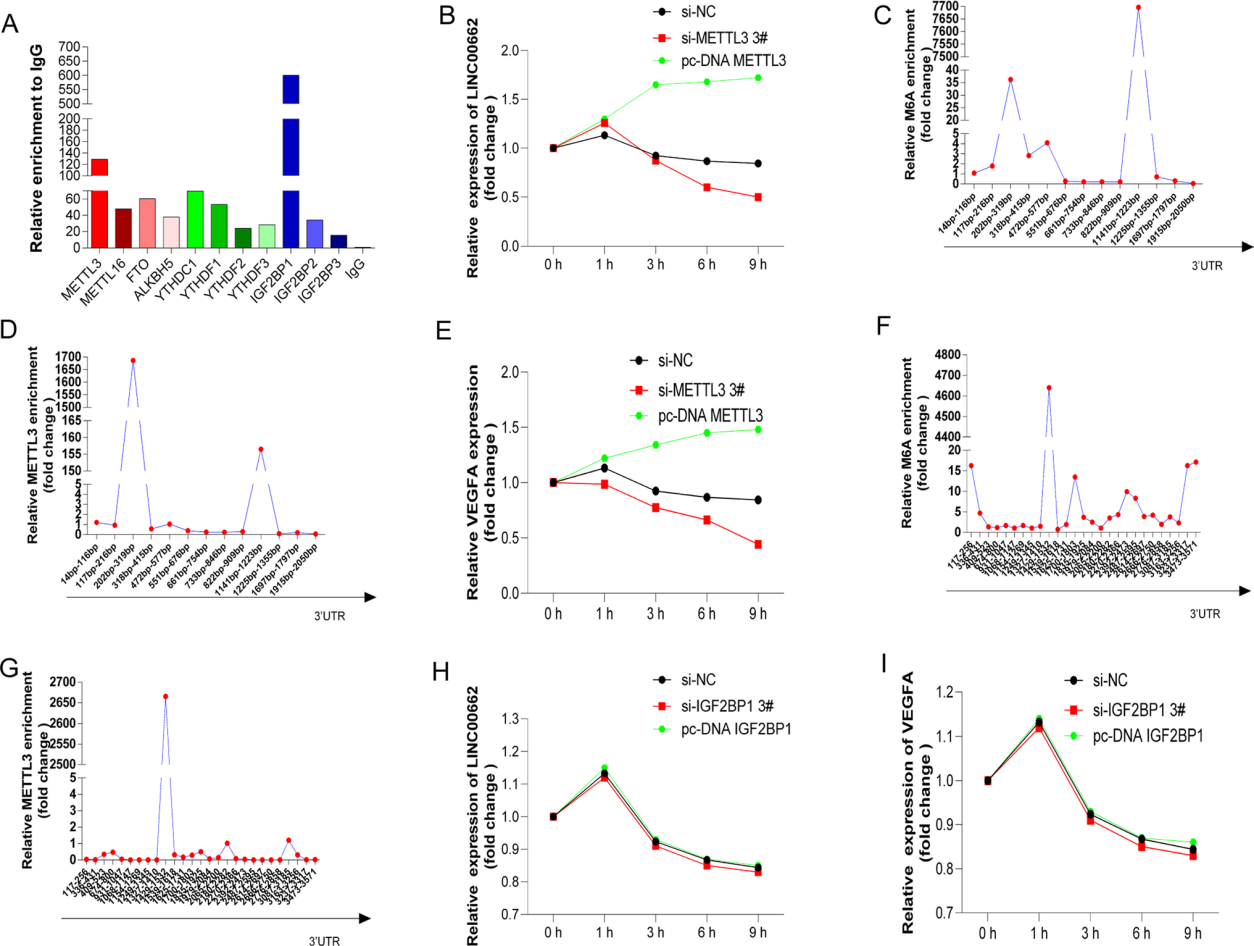
METTL3 dual regulation of LINC00662 and VEGFA RNAs stability. **A** RIP experiments verified the enrichment levels of LINC00662 and m6A enzymes. **B** Expression of LINC00662 RNA at 0 h, 1 h, 3 h, 6 h and 9 h after interference and overexpression of METTL3. **C** Validation of m6A enrichment level of LINC00662 RNA by MeRIP assay. **D** Validation of Mettl3 enrichment level of LINC00662 RNA by MeRIP assay. **E** Expression of VEGFA mRNA at 0 h, 1 h, 3 h, 6 h and 9 h after interference and overexpression of METTL3. **F** Validation of m6A enrichment level of VEGFA mRNA by MeRIP assay. **G** Validation of METTL3 enrichment level of VEGFA mRNA by MeRIP assay. **H** Expression of LINC00662 RNA at 0 h, 1 h, 3 h, 6 h and 9 h after interference and overexpression of IGF2BP1. **I** Expression of VEGFA mRNA at 0 h, 1 h, 3 h, 6 h and 9 h after interference and overexpression of IGF2BP1

The above study found that after interfering with METTL3, the LINC00662 and VEGFA RNAs were significantly down-regulated, and the protein level of VEGFA was also significantly down-regulated, suggesting that METTL3 could regulate the expression of LINC00662 and VEGFA. The above database prediction all showed that METTL3 could interact with LINC00662 ( Supplementary Fig. 1A). Therefore, the RIP experiment was first conducted in this project. M6A enzymes, including METTL3, METTL16, FTO, ALKBH5, YTHDC1, YTHDF1, YTHDF2, YTHDF3, IGF2BP1, IGF2BP2 and IGF2BP3, were incubated with HCT116 cell lysate at 4 °C. The qRT-PCR results of the pulled-down RNAs suggested that METTL3 and IGF2BP1 (insulin like growth factor 2 mRNA binding protein 1) could indeed enrich LINC00662 (Fig. 5A). Thus, it was clear that METTL3 and IGF2BP1 could interact with LINC00662. METTL3, as an m6A methyltransferase, primarily affected the stability of RNAs. After METTL3 was interfered and overexpressed, actinomycin was added to inhibit cell transcription, and the expression levels of the LINC00662 RNA in the cells were observed at 0 h, 1 h, 3 h, 6 h and 9 h (Fig. 5B). It was found that after METTL3 was interfered, the expression levels of the LINC00662 RNA decreased. Conversely, its expression increased. The MeRIP experiment (Fig. 5C, D) was then conducted in this study, and the results indicated that there were abundant m6A enrichment sites on the LINC00662 RNA. METTL3 was strongly enriched on the LINC00662 RNA, and it had interaction sites with LINC00662. These results confirmed that METTL3 could indeed affect the expression of LINC00662 by interacting with it to maintain its stability.

Following the same procedure, we observed the effect of METTL3 on the stability of VEGFA (Fig. 5E–G) and obtained results consistent with LINC00662. These results confirmed that METTL3 could indeed affect VEGFA expression by interacting with it to maintain its stability. In addition, the above database prediction (Supplementary Fig. 1A) and RIP results jointly suggested (Fig. 5A) that IGF2BP1 could also interact with LINC00662. The latest study found that IGF2BP1 also affected the stability of RNAs to regulate the expression of RNAs.

Therefore, we first analyzed the IGF2BP1 expression levels (Supplementary Fig. 2A) in the TCGA CRC database, and the results suggested that the IGF2BP1 mRNA was highly expressed in CRC. In the CPTAC protein prediction database (http://ualcan.path.uab.edu/analysis-prot.html), IGF2BP1 protein was also highly expressed (Supplementary Fig. 2B). Moreover, in 64 pairs of CRC and para-cancer tissues, IGF2BP1 mRNA was significantly higher in CRC compared with para-cancer tissues (Supplementary Fig. 2C, D). The IHC analysis also showed that the IGF2BP1 protein level was highly expressed in CRC (Supplementary Fig. 2E, F). Similarly, IGF2BP1 mRNA was significantly positively correlated with LINC00662 and VEGFA RNAs in 64 pairs of CRC tissues (Supplementary Fig. 2G, H). Subsequently, IGF2BP1 was disrupted in the HCT116 and HT29 cells, and it was found that LINC00662 and VEGFA RNAs were hardly down-regulated (Supplementary Fig. 2J, K). After interfering with IGF2BP1 and overexpressing it, the expression levels of the LINC00662 and VEGFA RNAs at 0 h, 1 h, 3 h, 6 h and 9 h were observed by adding actinomycin to inhibit cell transcription (Fig. 5H, I). The results showed that after interference with IGF2BP1 and the overexpression of it, no significant changes were observed in the LINC00662 and VEGFA RNAs, suggesting that IGF2BP1 had no effect on the stabilities of the LINC00662 and VEGFA RNAs. These results suggested that METTL3 could dually regulate the stabilities of the LINC00662 and VEGFA RNAs to maintain their high expressions.

### METTL3 and LINC00662 promoted the proliferation and migration of CRC cells

**Fig. 6:**
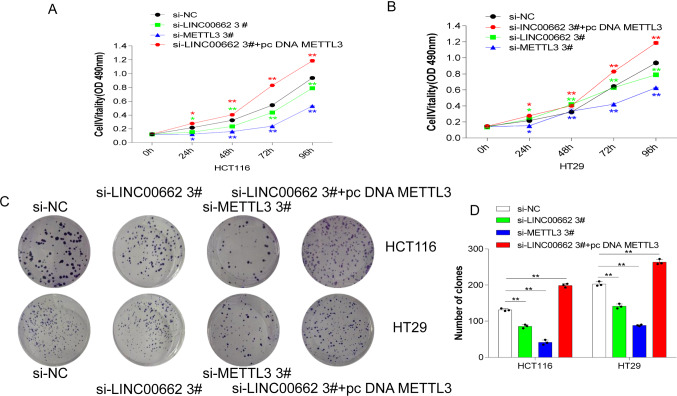
METTL3 and LINC00662 promoted proliferation of CRC cells. **A** Cell viabilities were detected by MTT assay after interference with si-NC, si-LINC00662 3#, si-METTL3 3# and si-LINC00662 3#+ pc DNA METTL3 in HCT116 cell. MTT was added at 0 h, 24 h, 48 h, 72 h and 96 h sequentially, and DMSO was added to detect the absorbance of cells at 490 nm. **B** Cell viabilities were detected by MTT assay after interference with si-NC, si-LINC00662 3#, si-METTL3 3# and si-LINC00662 3#+ pc DNA METTL3 in HT29 cell. MTT was added at 0 h, 24 h, 48 h, 72 h and 96 h sequentially, and DMSO was added to detect the absorbance of cells at 490 nm. **C** Cell clones were counted after interference with si-NC, si-LINC00662 3#, si-METTL3 3# and si-LINC00662 3#+ pc DNA METTL3 in HCT116 and HT29 cells. **D** Image Pro quantification plot of colony formation assay

It has been confirmed that METTL3 and LINC00662 can promote the angiogenesis of CRC cells. This topic was utilized to then study the biological functions of METTL3 and LINC00662 on cell proliferation and migration. Interfering with si-NC, si-LINC00662 3#, si-METTL3 3# and si-LINC00662 3#+ pc DNA METTL3 in HCT116 and HT29 cells, the proliferation function of the cells was observed. The MTT assay showed that the proliferation functions of the HCT116 and HT29 cells after interference with LINC00662 and METTL3 were obviously decreased. Compared with interfering LINC00662, interfering METTL3 could inhibit cell proliferation more significantly. It was worth noting that interfering with LINC00662 and overexpressing METTL3 could significantly reverse the inhibition of cell proliferation after interference with LINC00662 (Fig. 6A, B). The same was true for the results of colony formation assay (Fig. 6C, D). These results suggested that METTL3 and LINC00662 could promote the proliferation and migration of CRC cells HCT116 and HT29.

### METTL3 and LINC00662 promoted the proliferation and migration of CRC cells in zebrafish

**Fig. 7:**
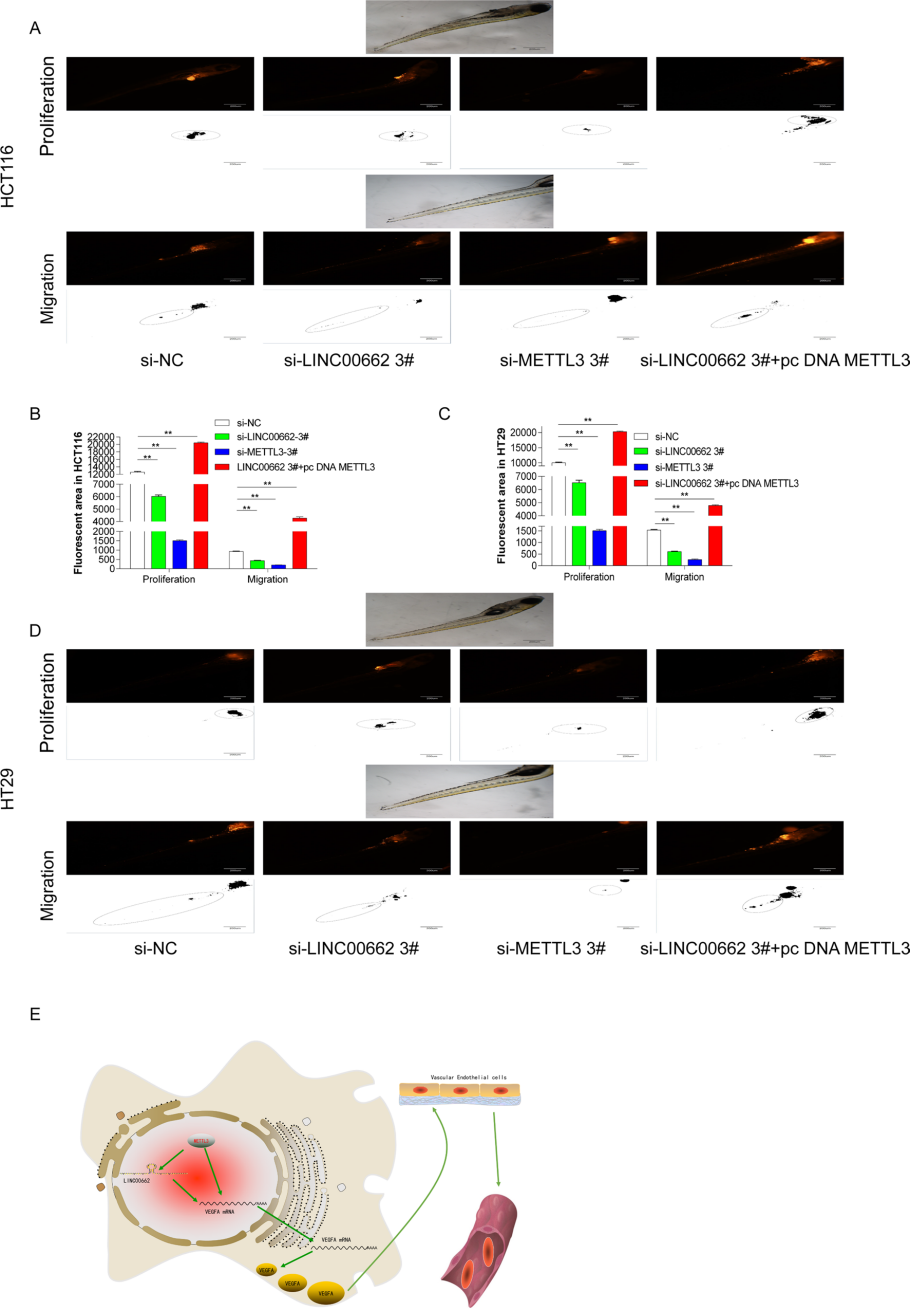
METTL3 and LINC00662 promoted the proliferation and migration of CRC cells in zebrafish. **A** Cell proliferation and migration assays in zebrafish after interference with si-NC, si-LINC00662 3#, si-METTL3 3# and si-LINC00662 3#+ pc DNA METTL3 in HCT116 cell. The images of proliferation and migration were taken by Olympus fluorescence microscope IX53 (Olympus, Center Valley, USA), and fluorescence area was calculated by Image J. **B** Fluorescence area of HCT116 cell proliferation and migration was quantified by Image J. **C** Fluorescence area of HT29 cell proliferation and migration was quantified by Image J. **D** Cell proliferation and migration assays in zebrafish after interference with si-NC, si-LINC00662 3#, si-METTL3 3# and si-LINC00662 3#+ pc DNA METTL3 in HT 29 cell. The images of proliferation and migration were taken by Olympus fluorescence microscope IX53 (Olympus, Center Valley, USA), and fluorescence area was calculated by Image J. **E** Mechanism diagram of METTL3 dual regulation of LINC00662 and VEGFA RNAs stability to promote angiogenesis in CRC

Previous results have confirmed that METTL3 and LINC00662 could promote the proliferation and migration of CRC cells HCT116 and HT29. In this study, zebrafish model animals were used to study the proliferation and migration functions of METTL3 and LINC00662 in vivo. A total of 24 h after interfering with the si-NC, si-LINC00662 3#, si-METTL3 3# and si-LINC00662 3#+ pc DNA the METTL3 in HCT116 cells, the cells were collected and washed twice with PBS at room temperature. They were then stained with yellow dye for 30 min in a 37 °C incubator medium and washed twice again. Finally, the cells were resuspended in 5 μl DMEM and injected into the yellow sac space and yellow sac space circulation system of the AB* juveniles incubated for 1 day. The images of proliferation and migration were taken by an Olympus fluorescence microscope IX53 (Olympus, Center Valley, PA, USA) (Fig. 7A), and the fluorescence area was calculated by Image J (Fig. 7B). The results suggested that the proliferation and migration functions of the HCT116 cells were significantly decreased after interference with LINC00662 and METTL3. Compared with interfering LINC00662, interfering METTL3 could inhibit cell proliferation and migration more obviously. It is worth noting that interfering with LINC00662 and overexpressing METTL3 significantly reversed the inhibitory effect on cell proliferation and migration after interfering with LINC00662. Next, we conducted the same experiment in the HT29 cells according to the above experimental steps, and obtained the same result as in the HCT116 cell (Fig. 7C, D). The above results demonstrated that METTL3 and LINC00662 could also promote the proliferation and migration of CRC cells HCT116 and HT29 in animals.

## Discussion

CRC is a common gastrointestinal malignancy with increasing incidence. The 5-year survival rate of patients with CRC is 65% [22], but the 5-year survival rate of patients with advanced CRC is extremely low. Therefore, technologies that help implement CRC treatment strategies are urgently required because they can improve patient survival [23]. It has been reported that the tumor microenvironment plays an obvious role in promoting the recurrence, drug resistance, and distal organ metastasis of CRC, especially angiogenesis. When tumors are larger than 2 mm, tumor angiogenesis greatly promotes the progression of CRC because blood is required to provide the needed oxygen and nutrients during tumor recurrence and metastasis. In recent years, studies on CRC angiogenesis have primarily focused on the transcriptional level [7–9]. Therefore, exploring new molecular mechanisms, especially regulation at the post-transcriptional level, will greatly enrich the study of CRC angiogenesis and provide a theoretical basis for colorectal diagnosis and treatment. Recent studies have found that m6A modification plays a key role in CRC [24–27]. In addition, m6A has also been reported to influence tumor progression by affecting angiogenesis [17, 28–32]. However, the effect of m6A on angiogenesis and progression remains unclear in CRC.

In this study, we first found a high expression of vascular-related indicators in CRC through the database, suggesting that angiogenesis may promote CRC progression. In the database analysis, we found that METTL3, the vascular-related m6A enzyme, was highly expressed in CRC tissues, indicating that METTL3 may promote the progression of CRC as well. We then confirmed that angiogenesis related indicators, such as VEGFA, are significantly overexpressed in CRC using qRT-PCR and IHC, confirming that angiogenesis could promote CRC progression. In addition, the IHC staining revealed a significantly high expression of m6A and METTL3 in CRC, suggesting that m6A modification, especially METTL3, may be indeed be involved in the progression of CRC. In addition, METTL3 was significantly positively correlated with VEGFA, and VEGFA mRNA and protein levels were significantly down-regulated after METTL3 interference, indicating that METTL3 may affect angiogenesis by regulating VEGFA in CRC.

With continuous studies on lncRNAs, it has become common to see that lncRNAs can promote the tumor microenvironment, especially angiogenesis, and thus can promote tumor progression [4, 20, 21]. The recent literature has found that METTL3 can promote the malignant progression of glioma by regulating the stability of MALAT1 and activating NF-κB [33]. In this study, we found LINC00662 through the CRC database. It was positively correlated with VEGFA. Further verification by histological qRT-PCR also showed that LINC00662 was highly expressed in CRC and was significantly positively correlated with VEGFA. This suggested that LINC00662 may promote CRC progression by influencing angiogenesis. It was worthy to note that qRT-PCR in CRC tissues also showed a significant positive correlation between METTL3 and LINC00662. After METTL3 interference, LINC00662 RNA levels decreased significantly, suggesting that METTL3 may regulate both LINC00662 and VEGFA RNAs to influence CRC angiogenesis.

Generally, m6A regulates the expression of genes by affecting the stability of its RNAs. In this study, the expression levels of the LINC00662 and VEGFA RNAs were observed separately after the interference and overexpression of METTL3. The results showed that after interference of METTL3 and the stabilities of LINC00662 and VEGFA RNAs were all decreased. Accordingly, the stabilities were increased after overexpression. Thus, the most important innovation of this study was that METTL3 could promote CRC angiogenesis by the dual regulation of LINC00662 and VEGFA RNAs stability.

M6A modification is primarily mediated by m6A methyltransferase, demethylase, and the reader protein that regulate pre-mRNA splicing, miRNA processing, translation and mRNA attenuation [34]. In this study, the RIP experiments showed that in addition to METTL3 interacting with LINC00662, the reader protein, IGF2BP1, also interacted with LINC00662. However, the stabilities and expression levels of LINC00662 and VEGFA RNAs did not change after the interference and overexpression of IGF2BP1. These results suggested that IGF2BP1 had no direct regulatory effect on LINC00662 and VEGFA. In addition, the overall survival of CRC patients with high METTL3 levels was shorter, which is consistent with those reported in other studies [26, 35]. Subsequently, we validated the role of METTL3 and LINC00662 in angiogenesis, proliferation, and migration in CRC. The knockdown of METTL3 could inhibit angiogenesis, proliferation, invasion, and migration in the HCT116 and HT29 cells. In addition, we established a multi-group rescue experiment in the studies of angiogenesis, proliferation, invasion, and the migration function. It showed that METTL3 played the most important role in promoting CRC angiogenesis through the METTL3/LINC00662/VEGFA axis, which greatly enriched the study of METTL3 on CRC angiogenesis. In addition, the theoretical basis of the m6A influence on the tumor microenvironment was strengthened. These results suggested that METTL3, as an oncogene, promoted the progression of CRC. The key role of METTL3 in various cancers has also been reported [33, 36, 37]. Therefore, METTL3 may become a new potential therapeutic target for cancer. In conclusion, our study suggests that METTL3 dually regulates the LINC00662 and VEGFA RNAs stability to promote CRC angiogenesis, which is a novel mechanism for regulating CRC angiogenesis. Although we demonstrated the regulatory mechanism of METTL3 in CRC angiogenesis, there were still deficiencies. For example, the selective primary catalytic inhibitor METTL3 (STM2457) has been reported as a therapeutic strategy for acute myeloid leukemia [38]. STM2457 combined with anti-PD-1 therapy also can achieve good anti-CRC tumor effect [39], but no METTL3 inhibitor has been found to be used for CRC anti-angiogenesis. Therefore, further studies are required to determine the role of METTL3 inhibitors in CRC angiogenesis. The failure of this study to construct stable cell lines with METTL3 knockdown to further confirm its role in the stability of LINC00662 and VEGFA RNAs is another deficiency of this paper.

## Conclusions

In conclusion, our study demonstrates that METTL3 can dually regulate the stability of the LINC00662 and VEGFA RNAs to promote the angiogenesis in CRC.METTL3 and LINC00662 may both serve as early diagnostic and prognostic predictive biomarkers for CRC and potential targets for anti-vascular therapy as well.

## Supplementary Information

**Additional file 1.** (ZIP 10940 KB)

**Additional file 2.** (DOCX 1165 KB)

## Data Availability

The original contributions presented in the study are included in the article/additional file. Further inquiries can be directed to the corresponding author.

## Abbreviations

CD31: Platelet endothelial cell adhesion molecule-1
CRC: Colorectal cancer
DEPC: Diethylpyrocarbonate
DMSO: Discarded and dimethyl sulfoxide
EDTA: Ethylene diamine tetraacetic acid
FBS: Fetal bovine serum
FHC: Fetal human colon
HUVEC: Human umbilical vein endothelial cells
IGF2BP1: Insulin-like growth factor 2 mRNA binding protein 1
IP: Immunoprecipitation
m6A: *N*6-Methyladenosine
METTL3: Methyltransferase-like 3
MeRIP: Methylated RNA immunoprecipitation
MTT: 3-(4,5)-Dimethylthiahiazo(-z-y1)-3,5-di-phenytetrazoliumromide
qRT-PCR: Quantitative real-time polymerase chain reaction
SDS: Sodium dodecyl sulfate
TCGA: The Cancer Genome Atlas
VEGFA: Vascular endothelial growth factor A
