# Supplementary figures and images for: METTL3 dual regulation of the stability of LINC00662 and VEGFA RNAs promotes colorectal cancer angiogenesis
Source: Discov Oncol. 2022 Sep 17;13:89. doi: 10.1007/s12672-022-00557-3 (PMC9482670; doi:10.1007/s12672-022-00557-3)

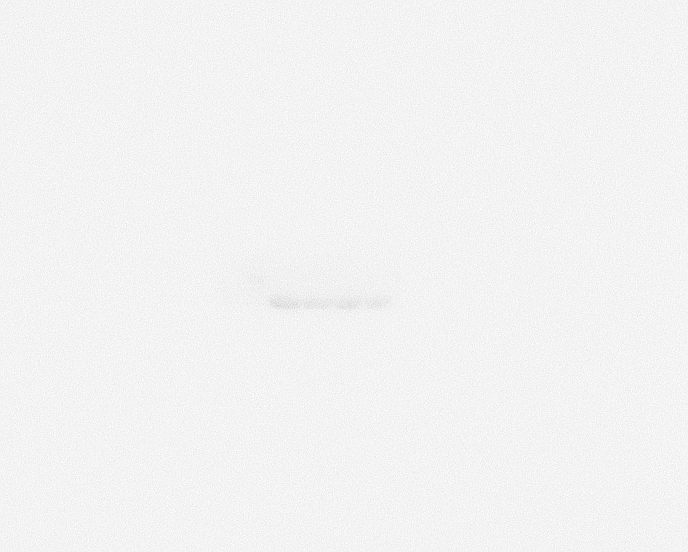

Supplement: Supplementary file 1 — Additional file 1. (ZIP 10940 KB) [file 12672_2022_557_MOESM1_ESM.zip › (1-2)/0.05s.tif]

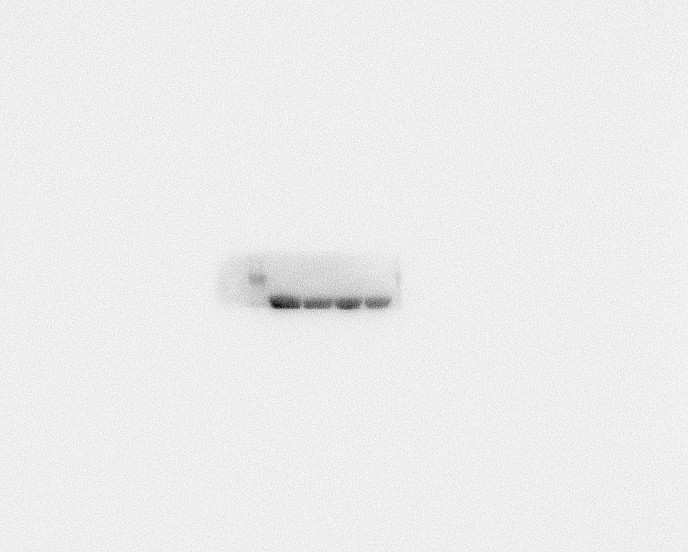

Supplement: Supplementary file 1 — Additional file 1. (ZIP 10940 KB) [file 12672_2022_557_MOESM1_ESM.zip › (1-2)/0.5s.tif]

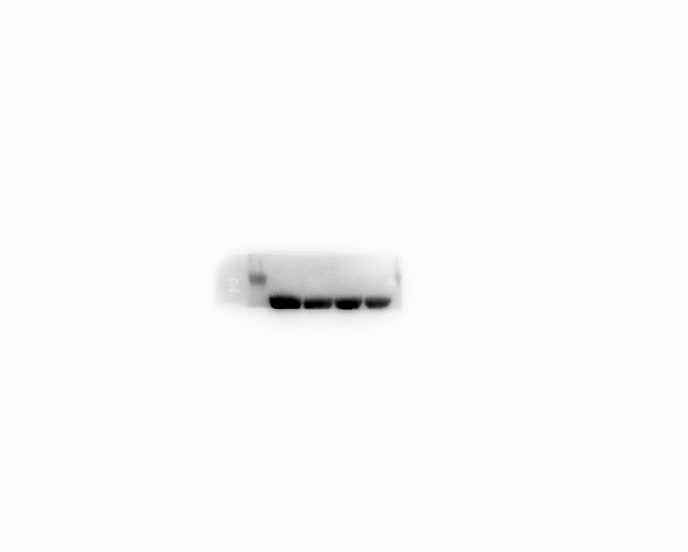

Supplement: Supplementary file 1 — Additional file 1. (ZIP 10940 KB) [file 12672_2022_557_MOESM1_ESM.zip › (1-2)/10s.tif]

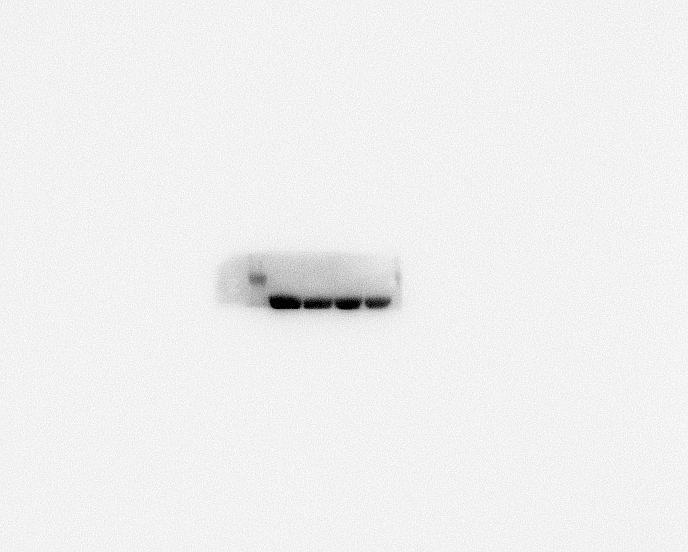

Supplement: Supplementary file 1 — Additional file 1. (ZIP 10940 KB) [file 12672_2022_557_MOESM1_ESM.zip › (1-2)/1s.tif]

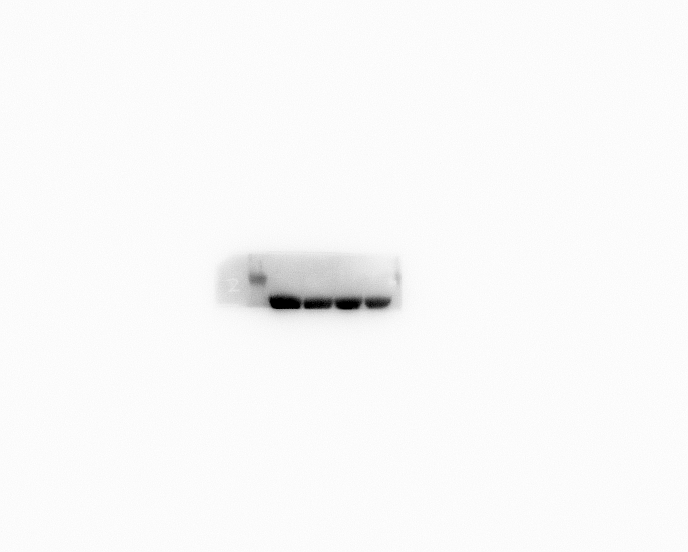

Supplement: Supplementary file 1 — Additional file 1. (ZIP 10940 KB) [file 12672_2022_557_MOESM1_ESM.zip › (1-2)/5s.tif]

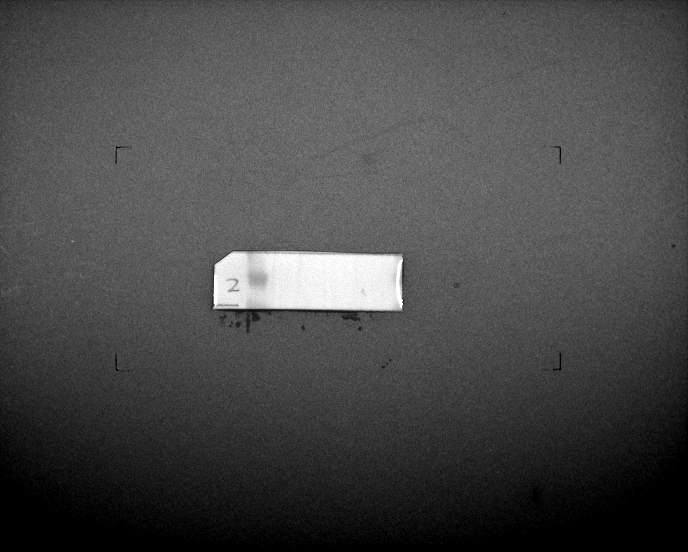

Supplement: Supplementary file 1 — Additional file 1. (ZIP 10940 KB) [file 12672_2022_557_MOESM1_ESM.zip › (1-2)/m.tif]

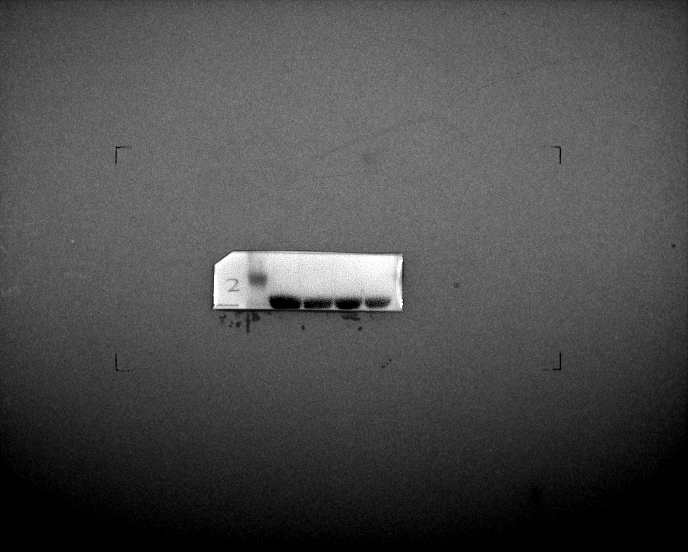

Supplement: Supplementary file 1 — Additional file 1. (ZIP 10940 KB) [file 12672_2022_557_MOESM1_ESM.zip › (1-2)/merge.tif]

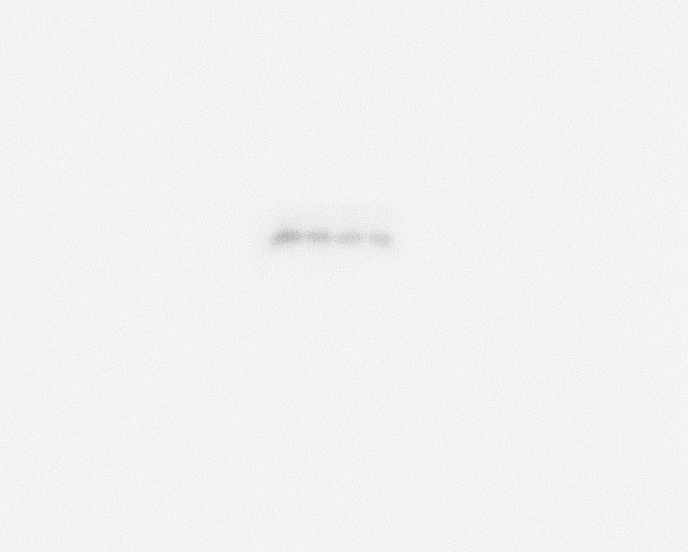

Supplement: Supplementary file 1 — Additional file 1. (ZIP 10940 KB) [file 12672_2022_557_MOESM1_ESM.zip › (1-12)/0.05s.tif]

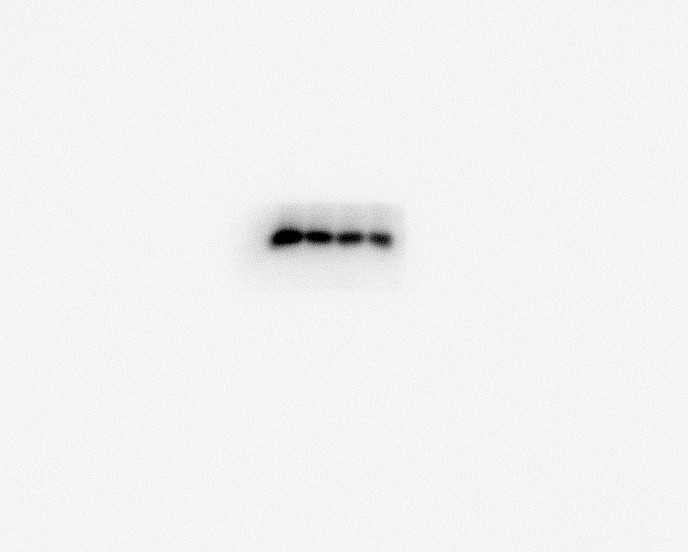

Supplement: Supplementary file 1 — Additional file 1. (ZIP 10940 KB) [file 12672_2022_557_MOESM1_ESM.zip › (1-12)/0.5s.tif]

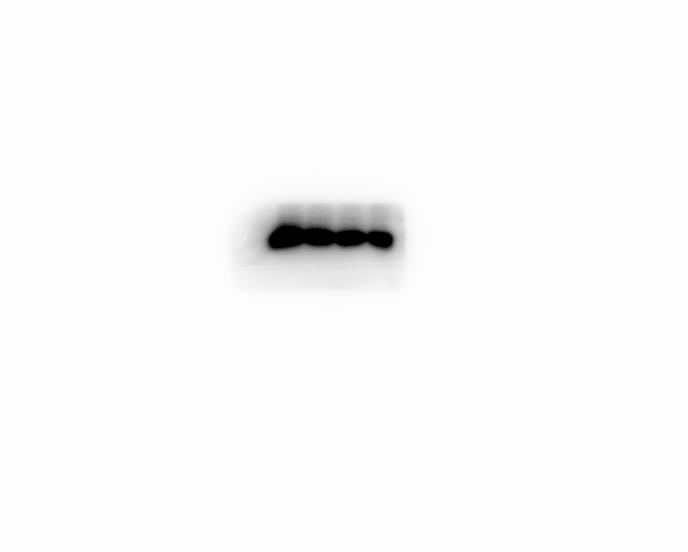

Supplement: Supplementary file 1 — Additional file 1. (ZIP 10940 KB) [file 12672_2022_557_MOESM1_ESM.zip › (1-12)/10s.tif]

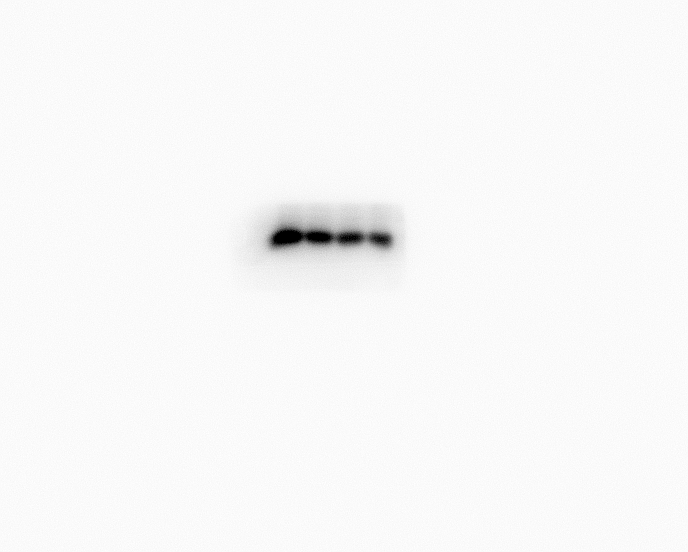

Supplement: Supplementary file 1 — Additional file 1. (ZIP 10940 KB) [file 12672_2022_557_MOESM1_ESM.zip › (1-12)/1s.tif]

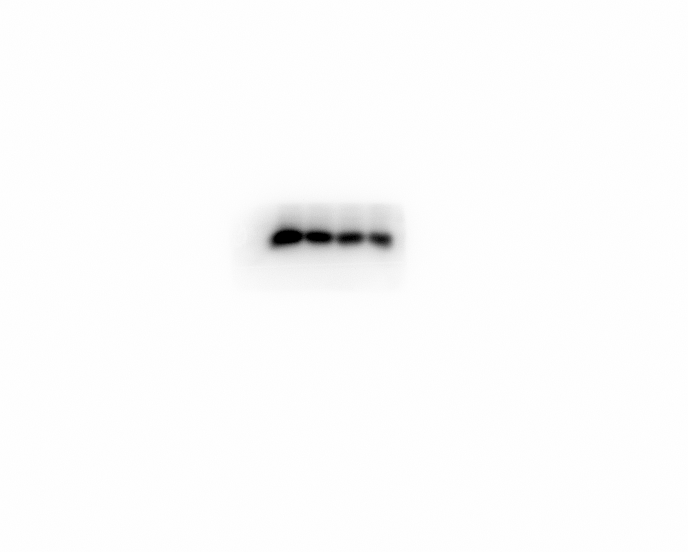

Supplement: Supplementary file 1 — Additional file 1. (ZIP 10940 KB) [file 12672_2022_557_MOESM1_ESM.zip › (1-12)/5s.tif]

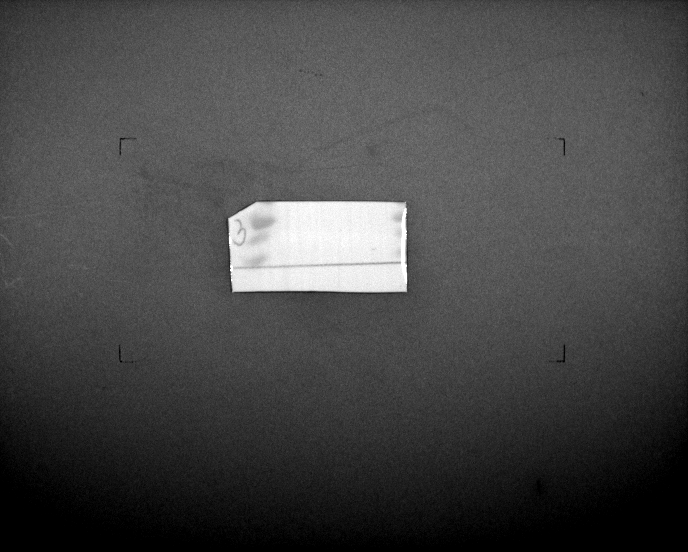

Supplement: Supplementary file 1 — Additional file 1. (ZIP 10940 KB) [file 12672_2022_557_MOESM1_ESM.zip › (1-12)/m.tif]

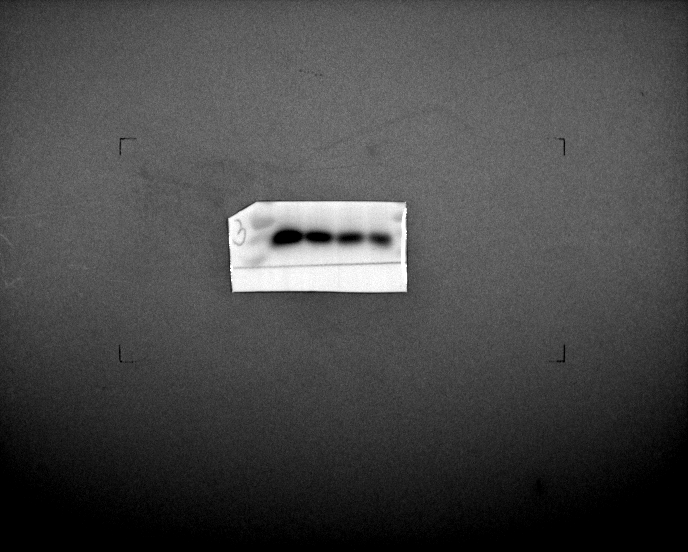

Supplement: Supplementary file 1 — Additional file 1. (ZIP 10940 KB) [file 12672_2022_557_MOESM1_ESM.zip › (1-12)/merge.tif]

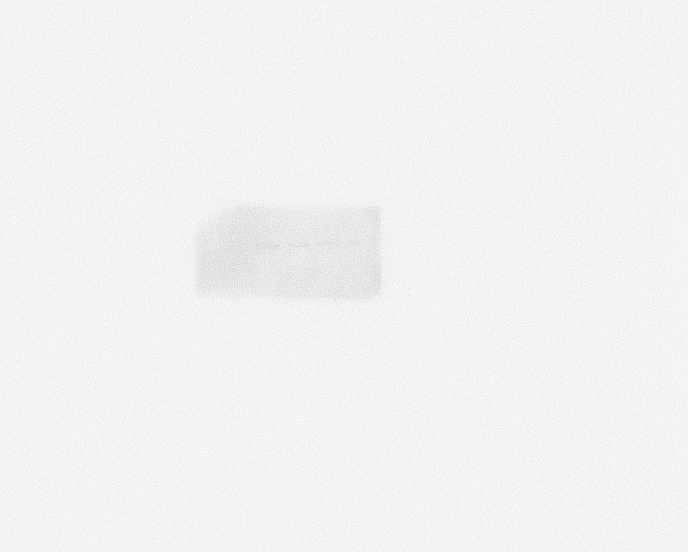

Supplement: Supplementary file 1 — Additional file 1. (ZIP 10940 KB) [file 12672_2022_557_MOESM1_ESM.zip › (1-4)/0.05s.tif]

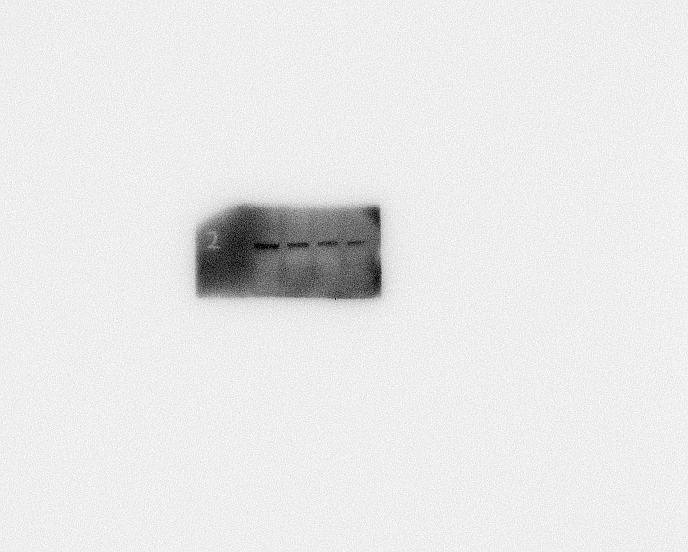

Supplement: Supplementary file 1 — Additional file 1. (ZIP 10940 KB) [file 12672_2022_557_MOESM1_ESM.zip › (1-4)/0.5s.tif]

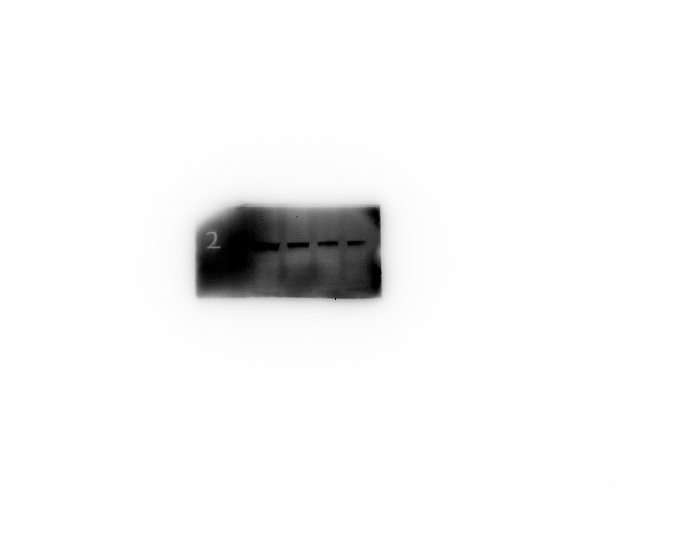

Supplement: Supplementary file 1 — Additional file 1. (ZIP 10940 KB) [file 12672_2022_557_MOESM1_ESM.zip › (1-4)/10s.tif]

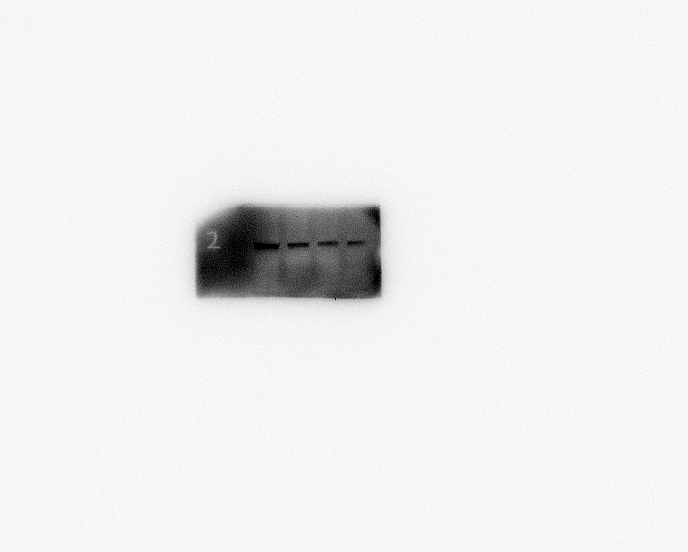

Supplement: Supplementary file 1 — Additional file 1. (ZIP 10940 KB) [file 12672_2022_557_MOESM1_ESM.zip › (1-4)/1s.tif]

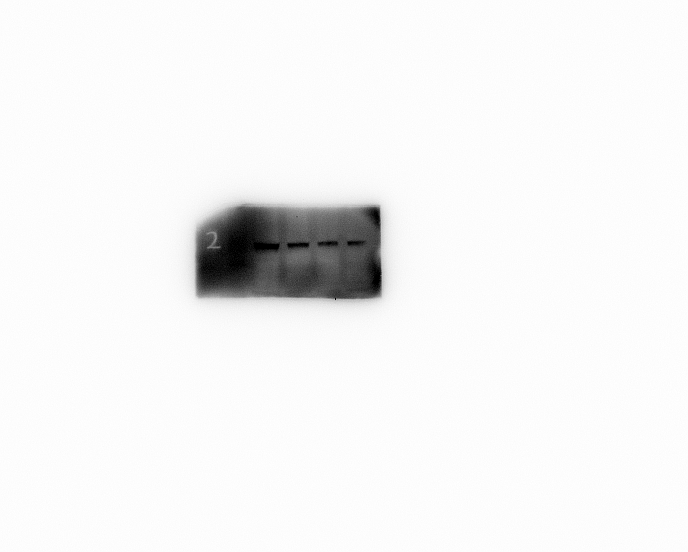

Supplement: Supplementary file 1 — Additional file 1. (ZIP 10940 KB) [file 12672_2022_557_MOESM1_ESM.zip › (1-4)/5s.tif]

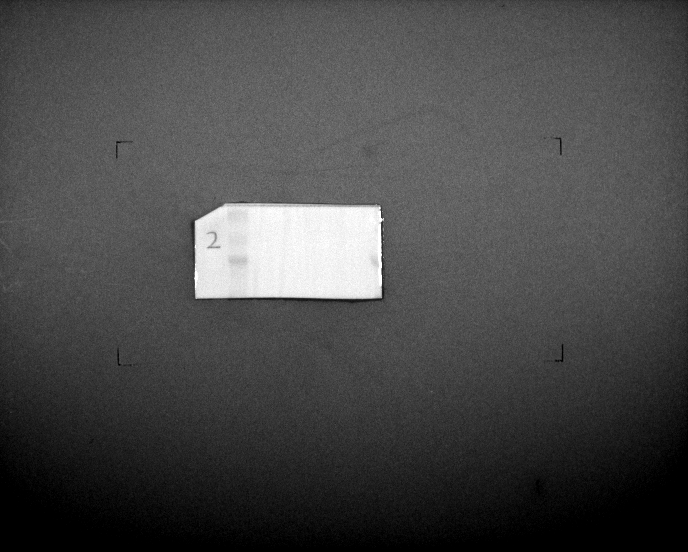

Supplement: Supplementary file 1 — Additional file 1. (ZIP 10940 KB) [file 12672_2022_557_MOESM1_ESM.zip › (1-4)/m.tif]

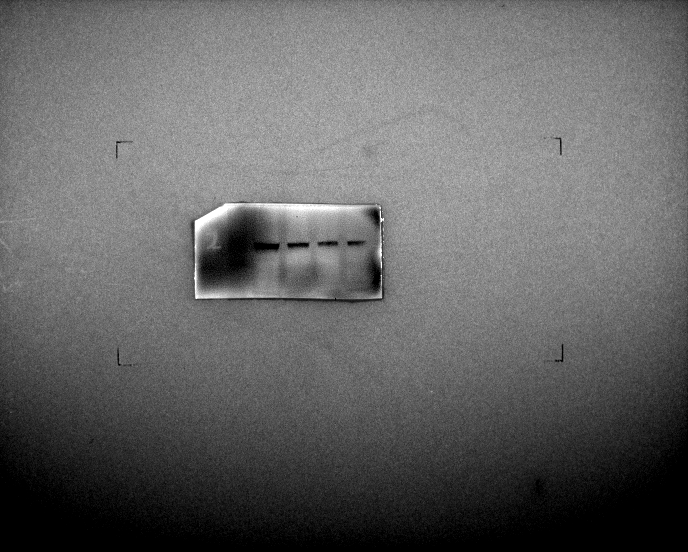

Supplement: Supplementary file 1 — Additional file 1. (ZIP 10940 KB) [file 12672_2022_557_MOESM1_ESM.zip › (1-4)/merge.tif]

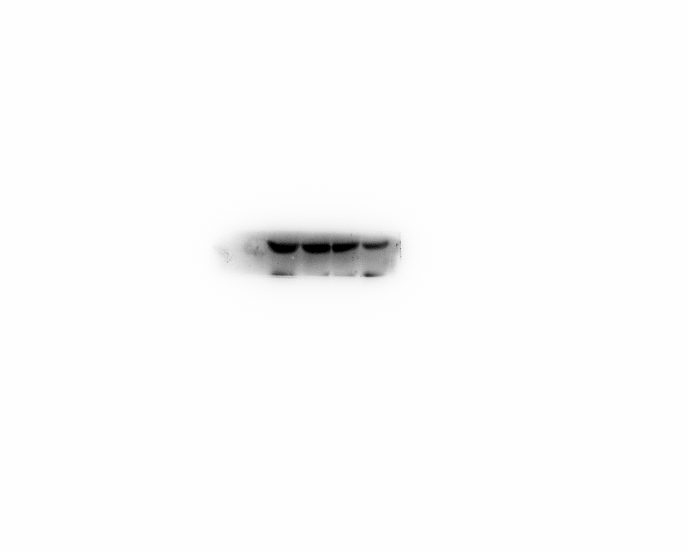

Supplement: Supplementary file 1 — Additional file 1. (ZIP 10940 KB) [file 12672_2022_557_MOESM1_ESM.zip › (1-5)/1min.tif]

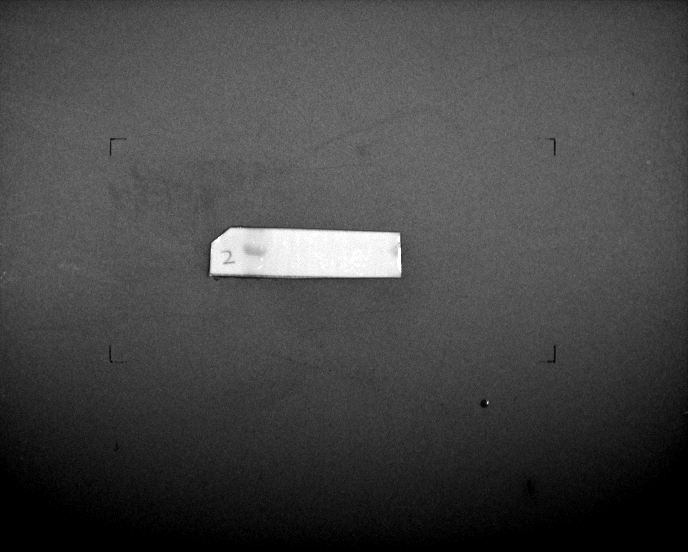

Supplement: Supplementary file 1 — Additional file 1. (ZIP 10940 KB) [file 12672_2022_557_MOESM1_ESM.zip › (1-5)/m.tif]

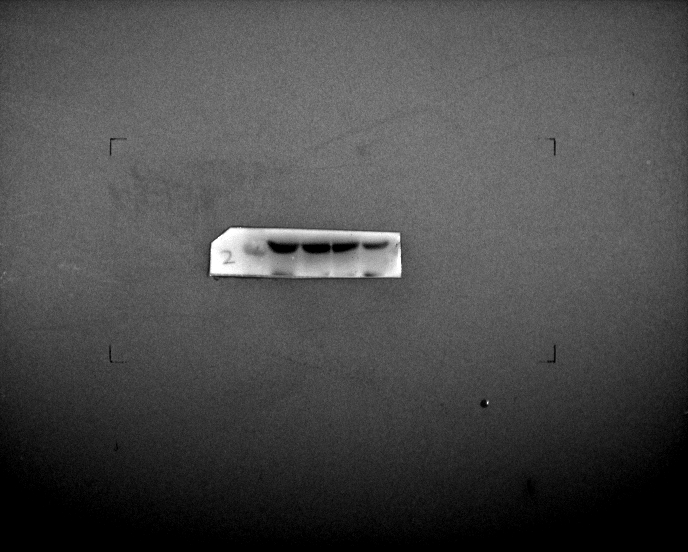

Supplement: Supplementary file 1 — Additional file 1. (ZIP 10940 KB) [file 12672_2022_557_MOESM1_ESM.zip › (1-5)/merge.tif]

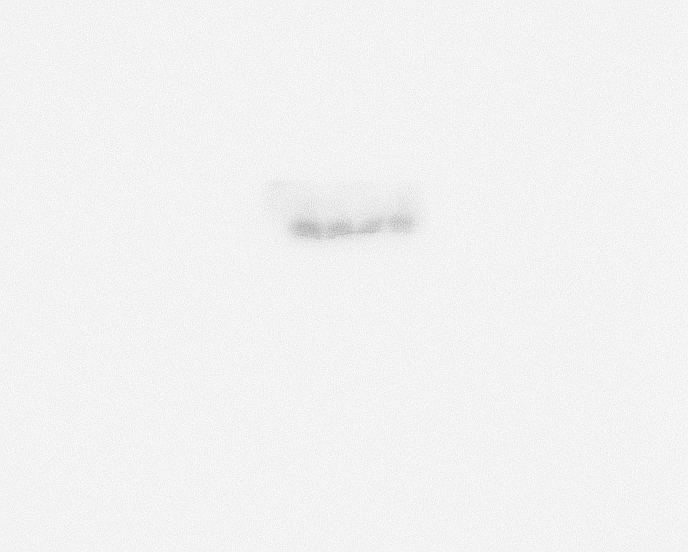

Supplement: Supplementary file 1 — Additional file 1. (ZIP 10940 KB) [file 12672_2022_557_MOESM1_ESM.zip › (1-6)/0.05s.tif]

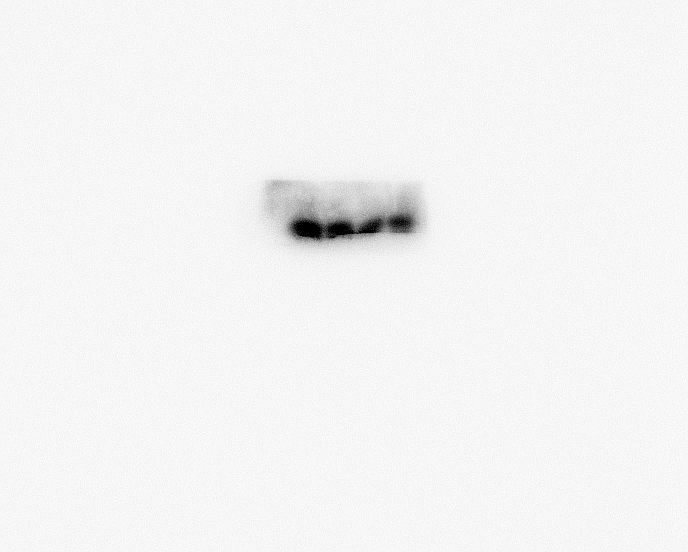

Supplement: Supplementary file 1 — Additional file 1. (ZIP 10940 KB) [file 12672_2022_557_MOESM1_ESM.zip › (1-6)/0.5s.tif]

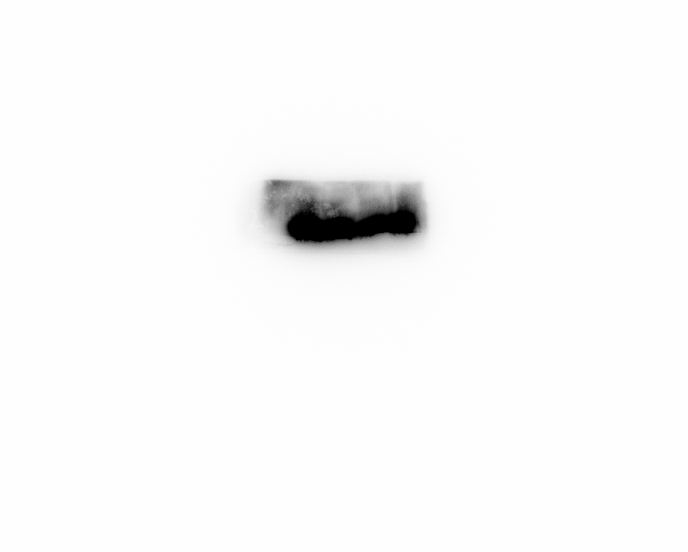

Supplement: Supplementary file 1 — Additional file 1. (ZIP 10940 KB) [file 12672_2022_557_MOESM1_ESM.zip › (1-6)/10s.tif]

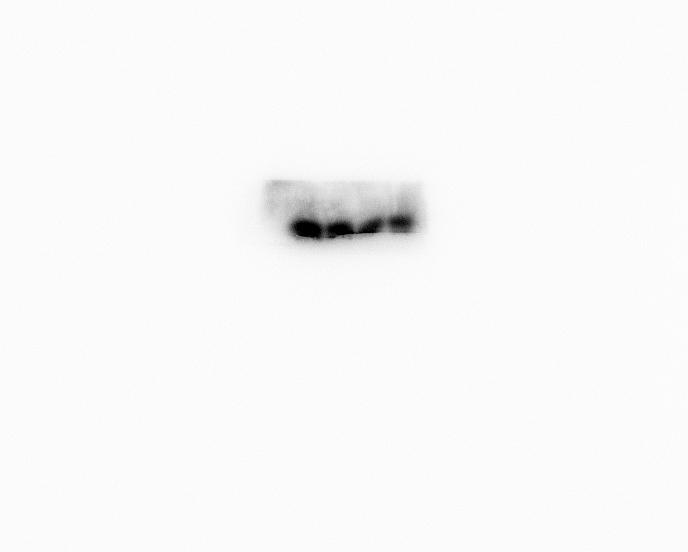

Supplement: Supplementary file 1 — Additional file 1. (ZIP 10940 KB) [file 12672_2022_557_MOESM1_ESM.zip › (1-6)/1s.tif]

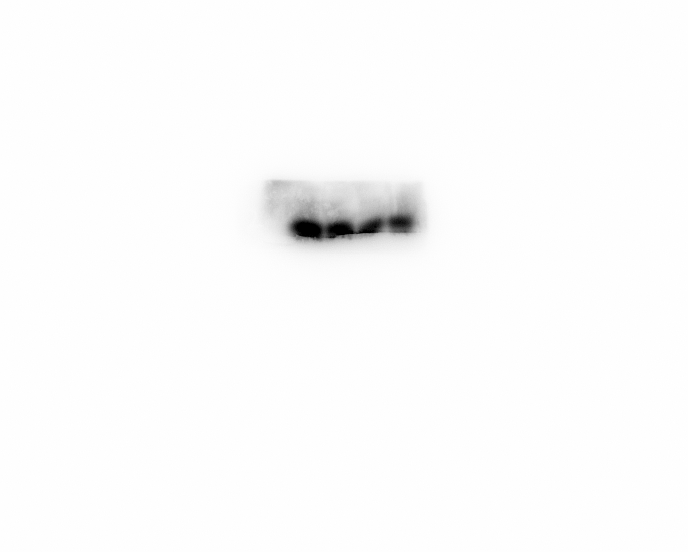

Supplement: Supplementary file 1 — Additional file 1. (ZIP 10940 KB) [file 12672_2022_557_MOESM1_ESM.zip › (1-6)/5s.tif]

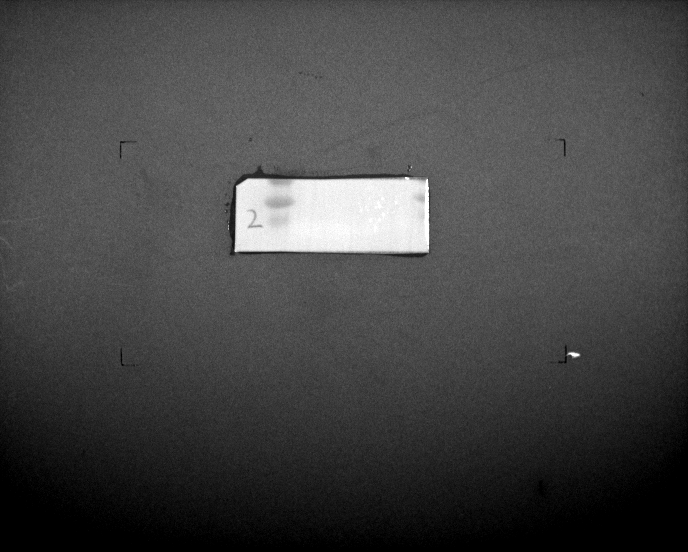

Supplement: Supplementary file 1 — Additional file 1. (ZIP 10940 KB) [file 12672_2022_557_MOESM1_ESM.zip › (1-6)/m.tif]

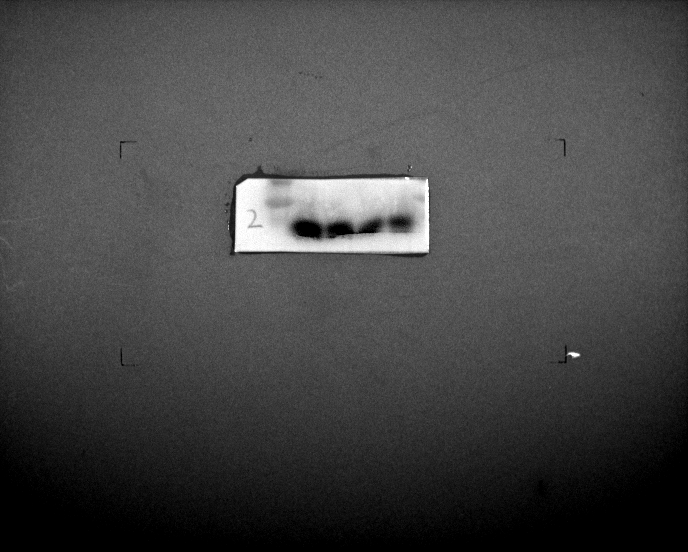

Supplement: Supplementary file 1 — Additional file 1. (ZIP 10940 KB) [file 12672_2022_557_MOESM1_ESM.zip › (1-6)/merge.tif]

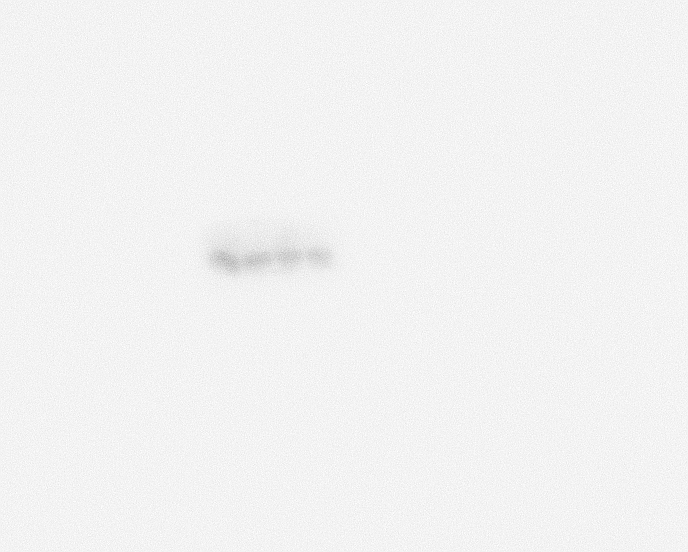

Supplement: Supplementary file 1 — Additional file 1. (ZIP 10940 KB) [file 12672_2022_557_MOESM1_ESM.zip › (1-7)/0.05s.tif]

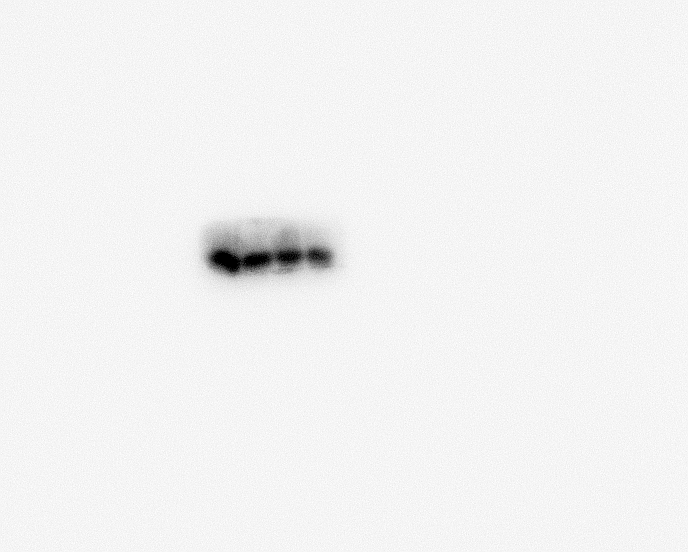

Supplement: Supplementary file 1 — Additional file 1. (ZIP 10940 KB) [file 12672_2022_557_MOESM1_ESM.zip › (1-7)/0.5s.tif]

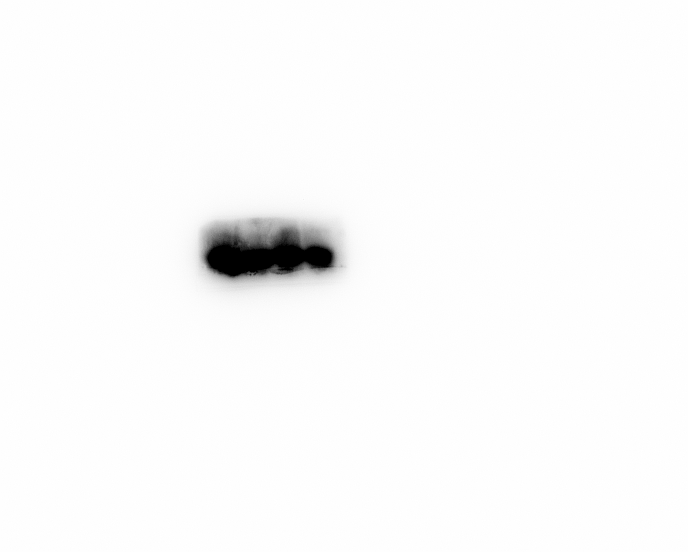

Supplement: Supplementary file 1 — Additional file 1. (ZIP 10940 KB) [file 12672_2022_557_MOESM1_ESM.zip › (1-7)/10s.tif]

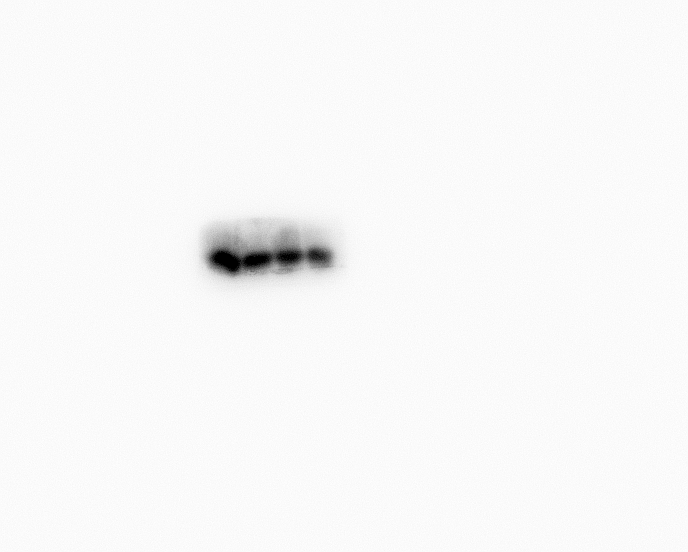

Supplement: Supplementary file 1 — Additional file 1. (ZIP 10940 KB) [file 12672_2022_557_MOESM1_ESM.zip › (1-7)/1s.tif]

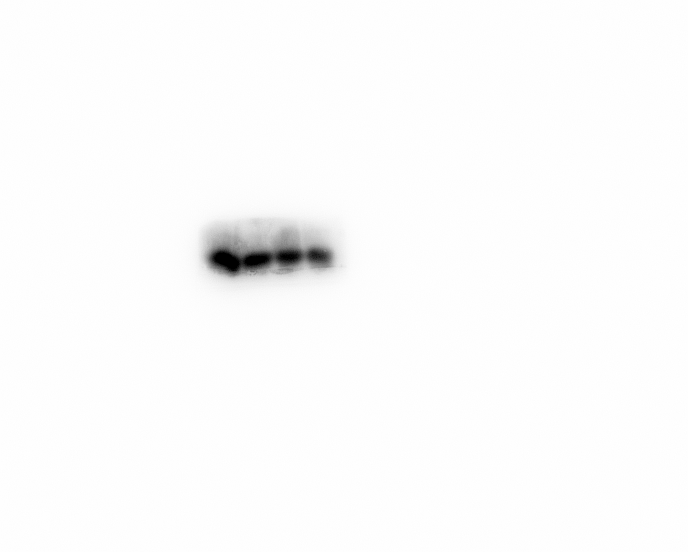

Supplement: Supplementary file 1 — Additional file 1. (ZIP 10940 KB) [file 12672_2022_557_MOESM1_ESM.zip › (1-7)/5s.tif]

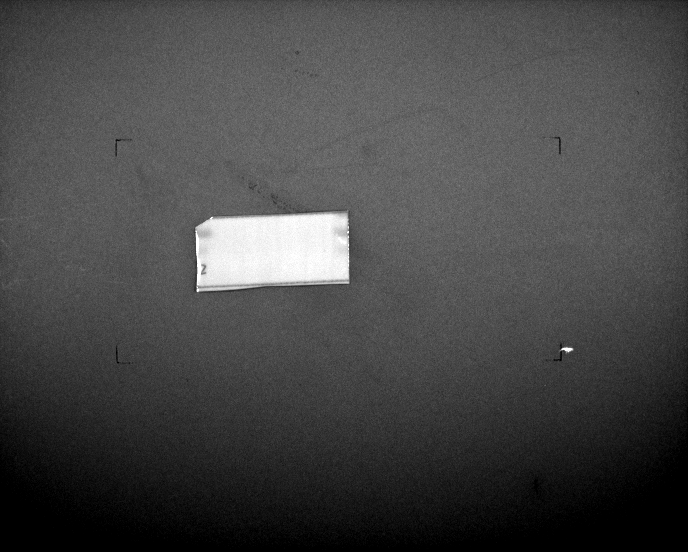

Supplement: Supplementary file 1 — Additional file 1. (ZIP 10940 KB) [file 12672_2022_557_MOESM1_ESM.zip › (1-7)/m.tif]

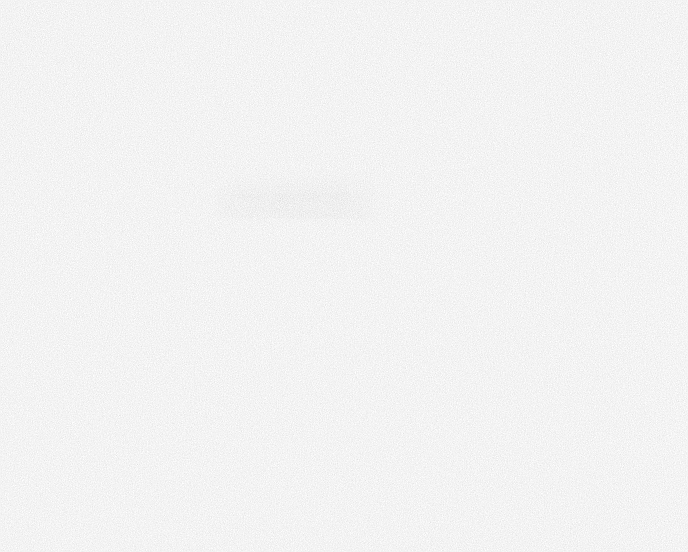

Supplement: Supplementary file 1 — Additional file 1. (ZIP 10940 KB) [file 12672_2022_557_MOESM1_ESM.zip › (3-2)/0.05s.tif]

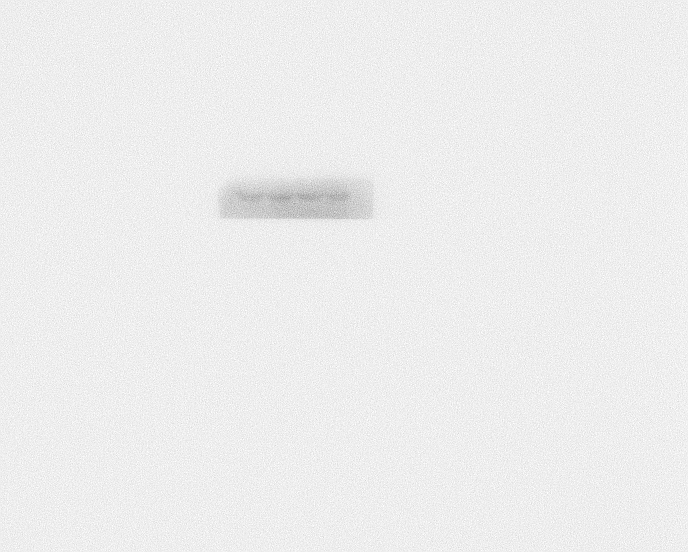

Supplement: Supplementary file 1 — Additional file 1. (ZIP 10940 KB) [file 12672_2022_557_MOESM1_ESM.zip › (3-2)/0.5s.tif]

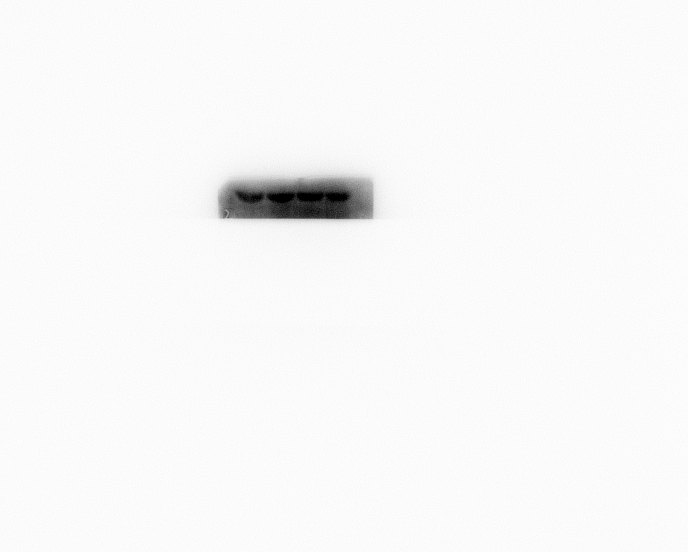

Supplement: Supplementary file 1 — Additional file 1. (ZIP 10940 KB) [file 12672_2022_557_MOESM1_ESM.zip › (3-2)/10s.tif]

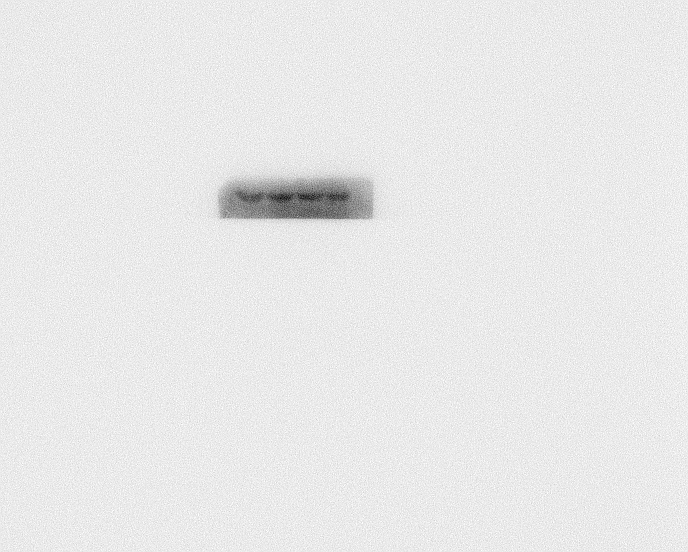

Supplement: Supplementary file 1 — Additional file 1. (ZIP 10940 KB) [file 12672_2022_557_MOESM1_ESM.zip › (3-2)/1s.tif]

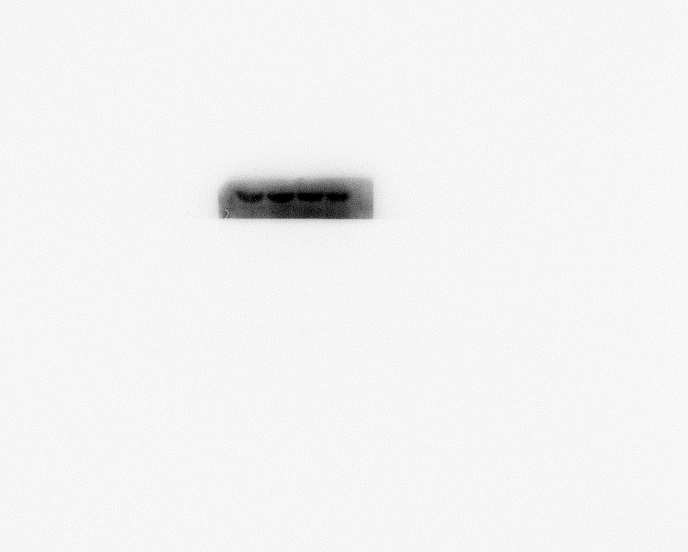

Supplement: Supplementary file 1 — Additional file 1. (ZIP 10940 KB) [file 12672_2022_557_MOESM1_ESM.zip › (3-2)/5s.tif]

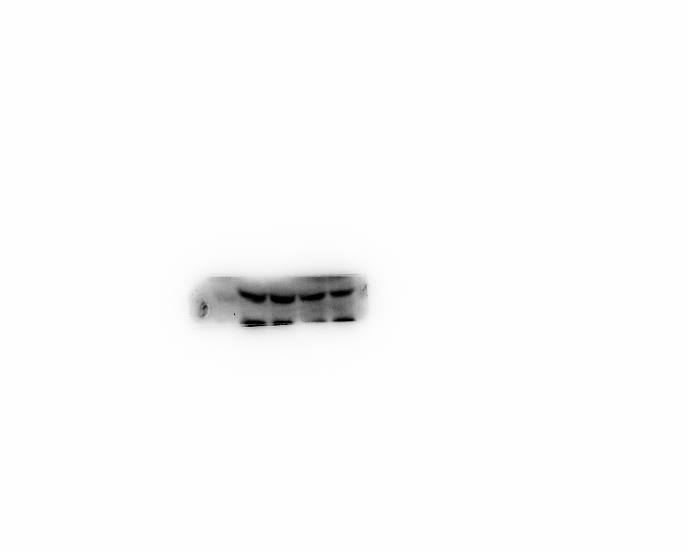

Supplement: Supplementary file 1 — Additional file 1. (ZIP 10940 KB) [file 12672_2022_557_MOESM1_ESM.zip › (3-3)/1min.tif]

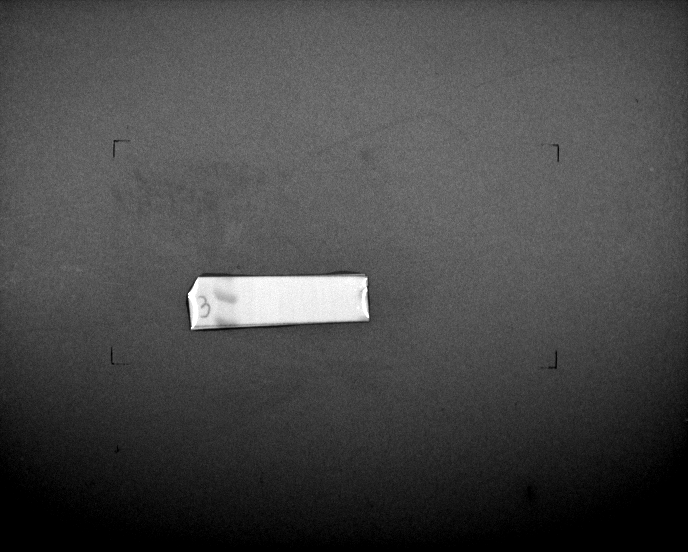

Supplement: Supplementary file 1 — Additional file 1. (ZIP 10940 KB) [file 12672_2022_557_MOESM1_ESM.zip › (3-3)/m.tif]

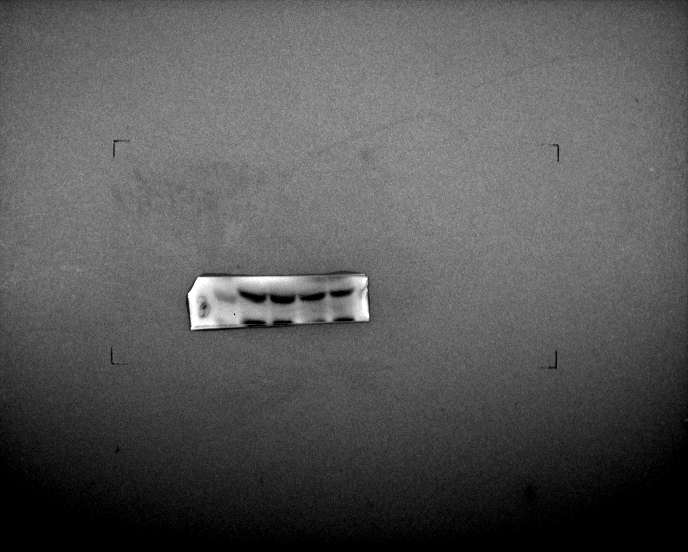

Supplement: Supplementary file 1 — Additional file 1. (ZIP 10940 KB) [file 12672_2022_557_MOESM1_ESM.zip › (3-3)/merge.tif]

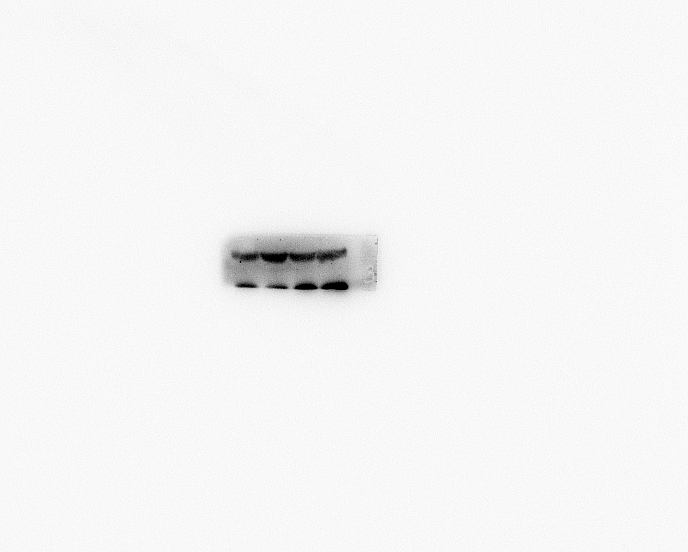

Supplement: Supplementary file 1 — Additional file 1. (ZIP 10940 KB) [file 12672_2022_557_MOESM1_ESM.zip › (3-4)/10s.tif]

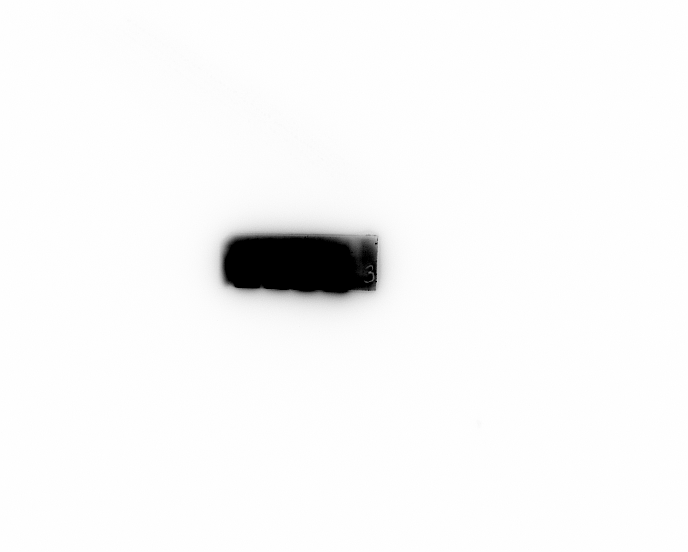

Supplement: Supplementary file 1 — Additional file 1. (ZIP 10940 KB) [file 12672_2022_557_MOESM1_ESM.zip › (3-4)/120s.tif]

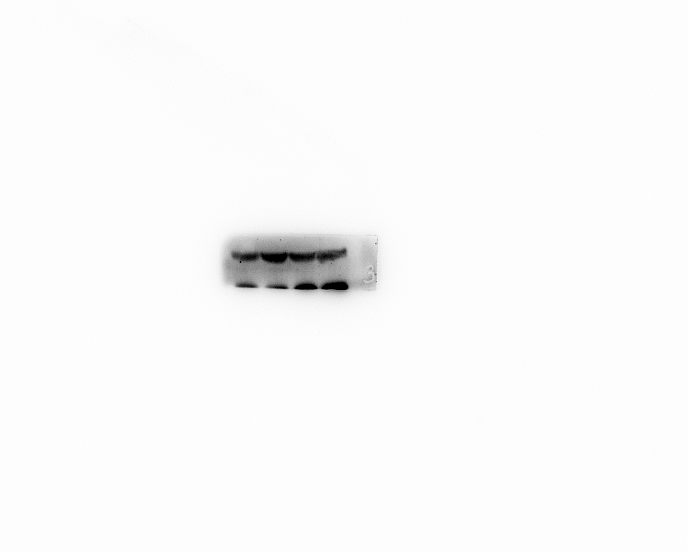

Supplement: Supplementary file 1 — Additional file 1. (ZIP 10940 KB) [file 12672_2022_557_MOESM1_ESM.zip › (3-4)/30s.tif]

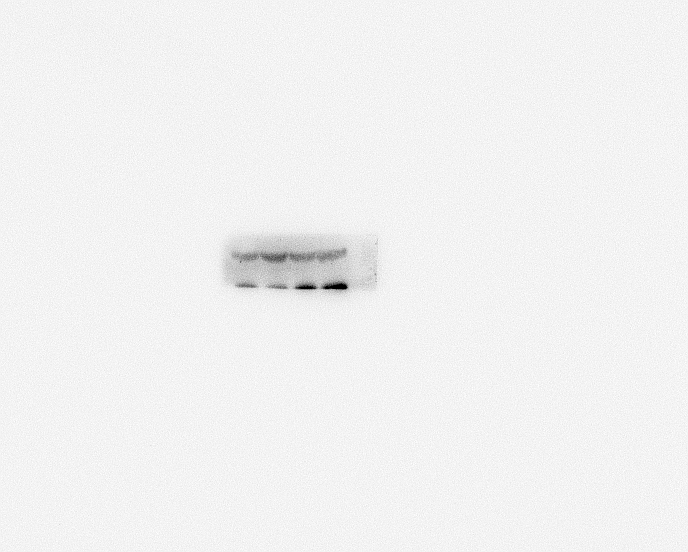

Supplement: Supplementary file 1 — Additional file 1. (ZIP 10940 KB) [file 12672_2022_557_MOESM1_ESM.zip › (3-4)/3s.tif]

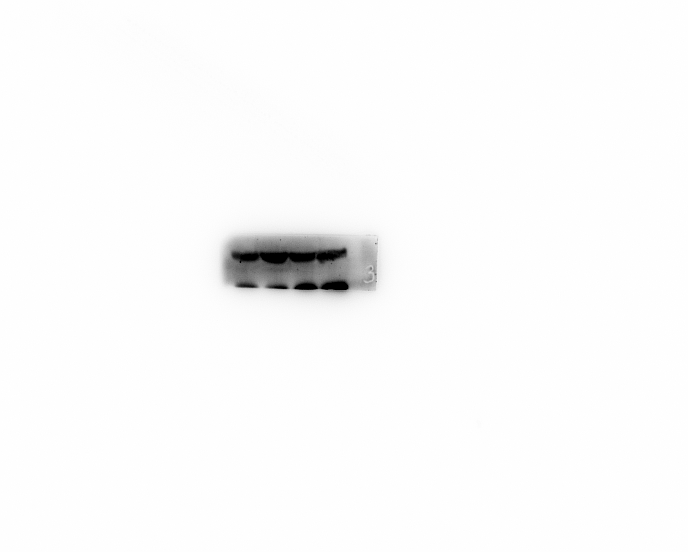

Supplement: Supplementary file 1 — Additional file 1. (ZIP 10940 KB) [file 12672_2022_557_MOESM1_ESM.zip › (3-4)/60s.tif]

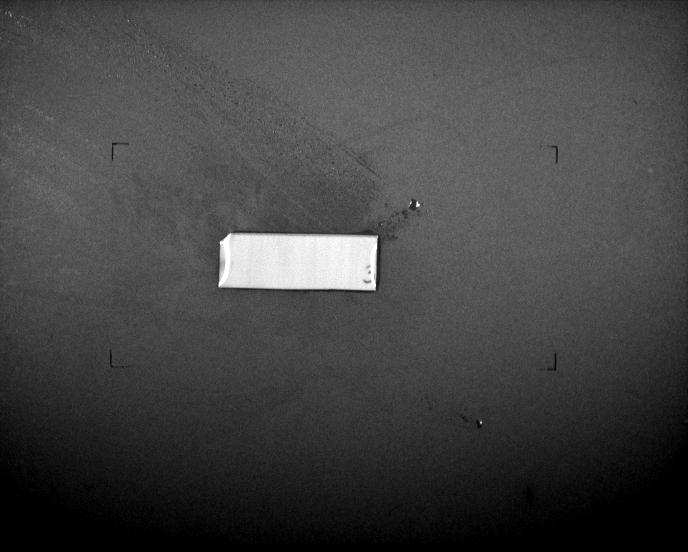

Supplement: Supplementary file 1 — Additional file 1. (ZIP 10940 KB) [file 12672_2022_557_MOESM1_ESM.zip › (3-4)/m.tif]

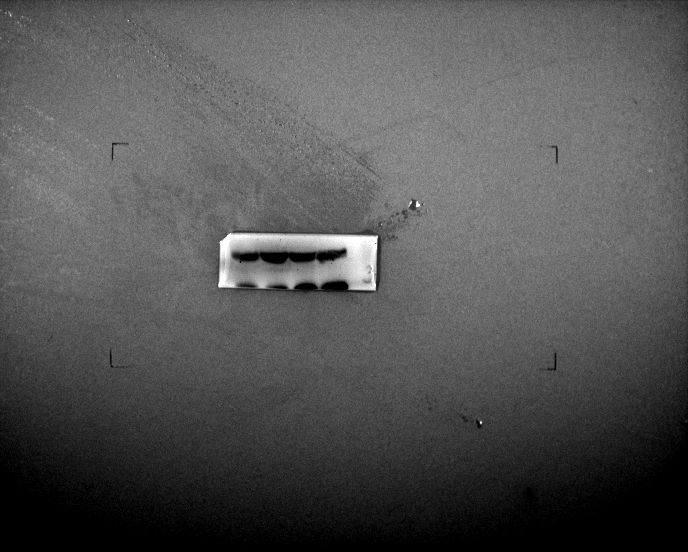

Supplement: Supplementary file 1 — Additional file 1. (ZIP 10940 KB) [file 12672_2022_557_MOESM1_ESM.zip › (3-4)/merge.tif]

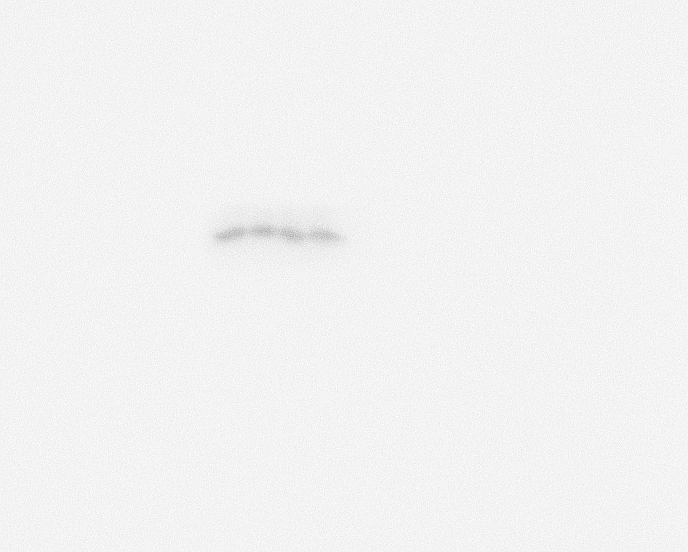

Supplement: Supplementary file 1 — Additional file 1. (ZIP 10940 KB) [file 12672_2022_557_MOESM1_ESM.zip › (3-5)/0.05s.tif]

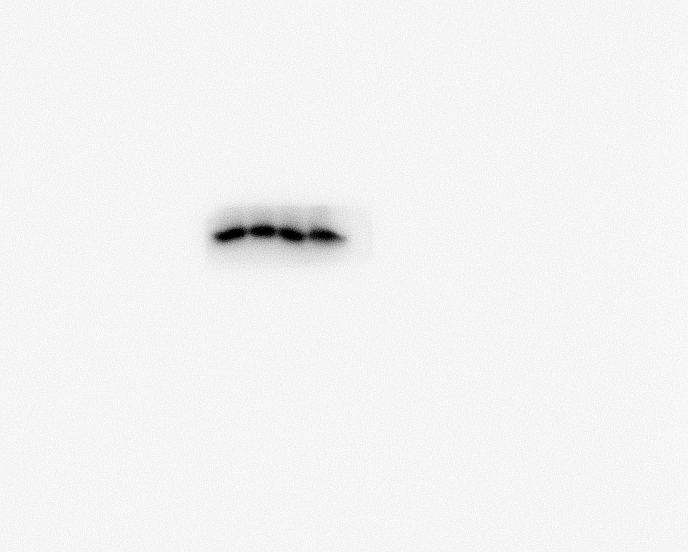

Supplement: Supplementary file 1 — Additional file 1. (ZIP 10940 KB) [file 12672_2022_557_MOESM1_ESM.zip › (3-5)/0.5s.tif]

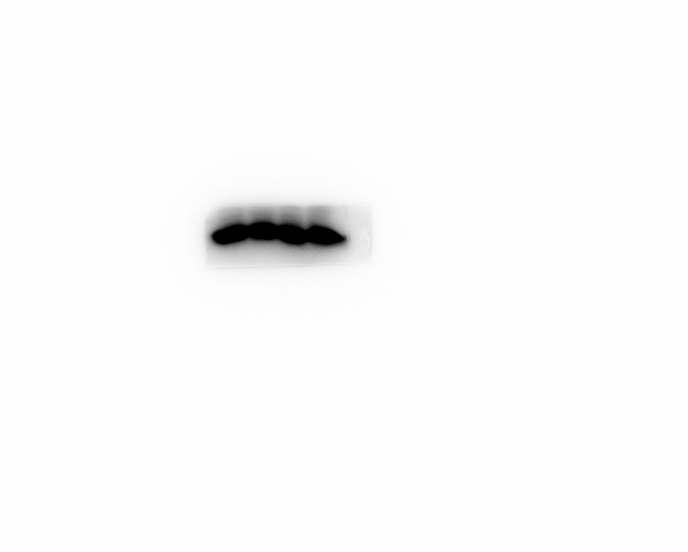

Supplement: Supplementary file 1 — Additional file 1. (ZIP 10940 KB) [file 12672_2022_557_MOESM1_ESM.zip › (3-5)/10s.tif]

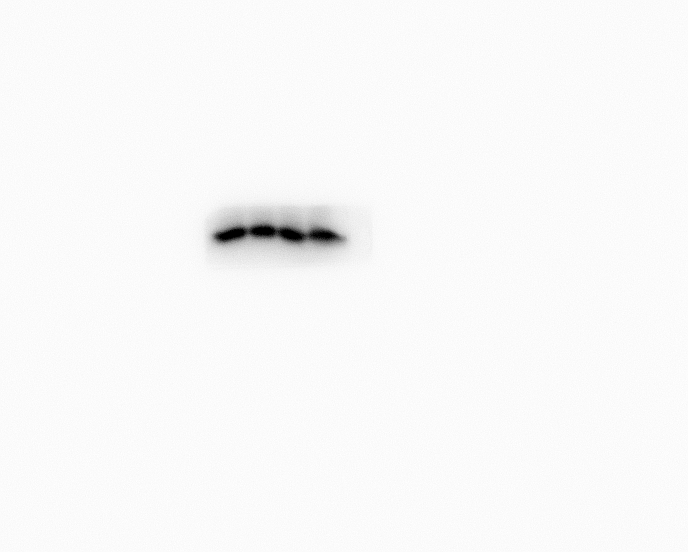

Supplement: Supplementary file 1 — Additional file 1. (ZIP 10940 KB) [file 12672_2022_557_MOESM1_ESM.zip › (3-5)/1s.tif]

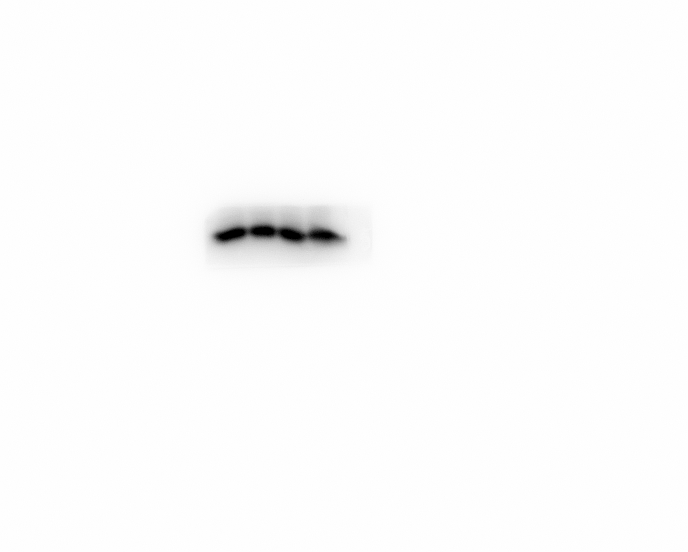

Supplement: Supplementary file 1 — Additional file 1. (ZIP 10940 KB) [file 12672_2022_557_MOESM1_ESM.zip › (3-5)/5s.tif]

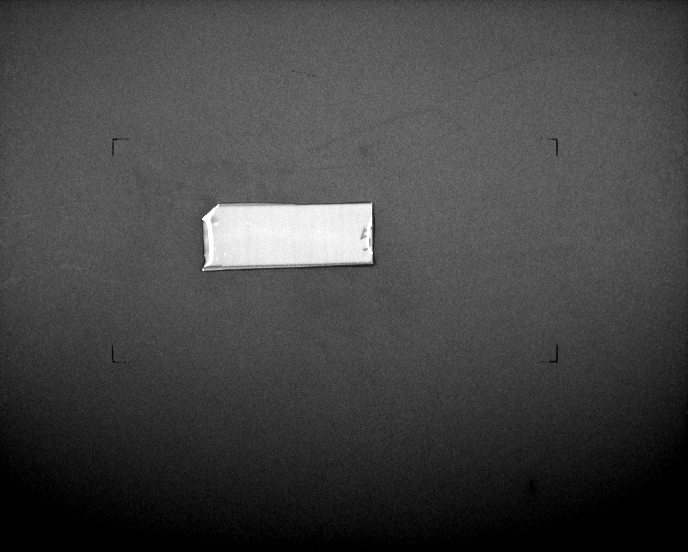

Supplement: Supplementary file 1 — Additional file 1. (ZIP 10940 KB) [file 12672_2022_557_MOESM1_ESM.zip › (3-5)/m.tif]

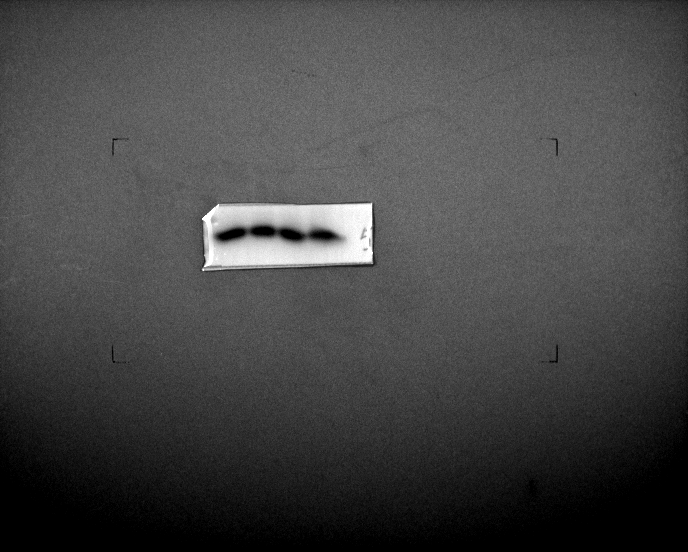

Supplement: Supplementary file 1 — Additional file 1. (ZIP 10940 KB) [file 12672_2022_557_MOESM1_ESM.zip › (3-5)/merge.tif]
