# Supplementary material for: METTL3 dual regulation of the stability of LINC00662 and VEGFA RNAs promotes colorectal cancer angiogenesis
Source: Discov Oncol. 2022 Sep 17;13:89. doi: 10.1007/s12672-022-00557-3 (PMC9482670; doi:10.1007/s12672-022-00557-3)
Supplement: Supplementary file 2 — Additional file 2. (DOCX 1165 KB) [file 12672_2022_557_MOESM2_ESM.docx]

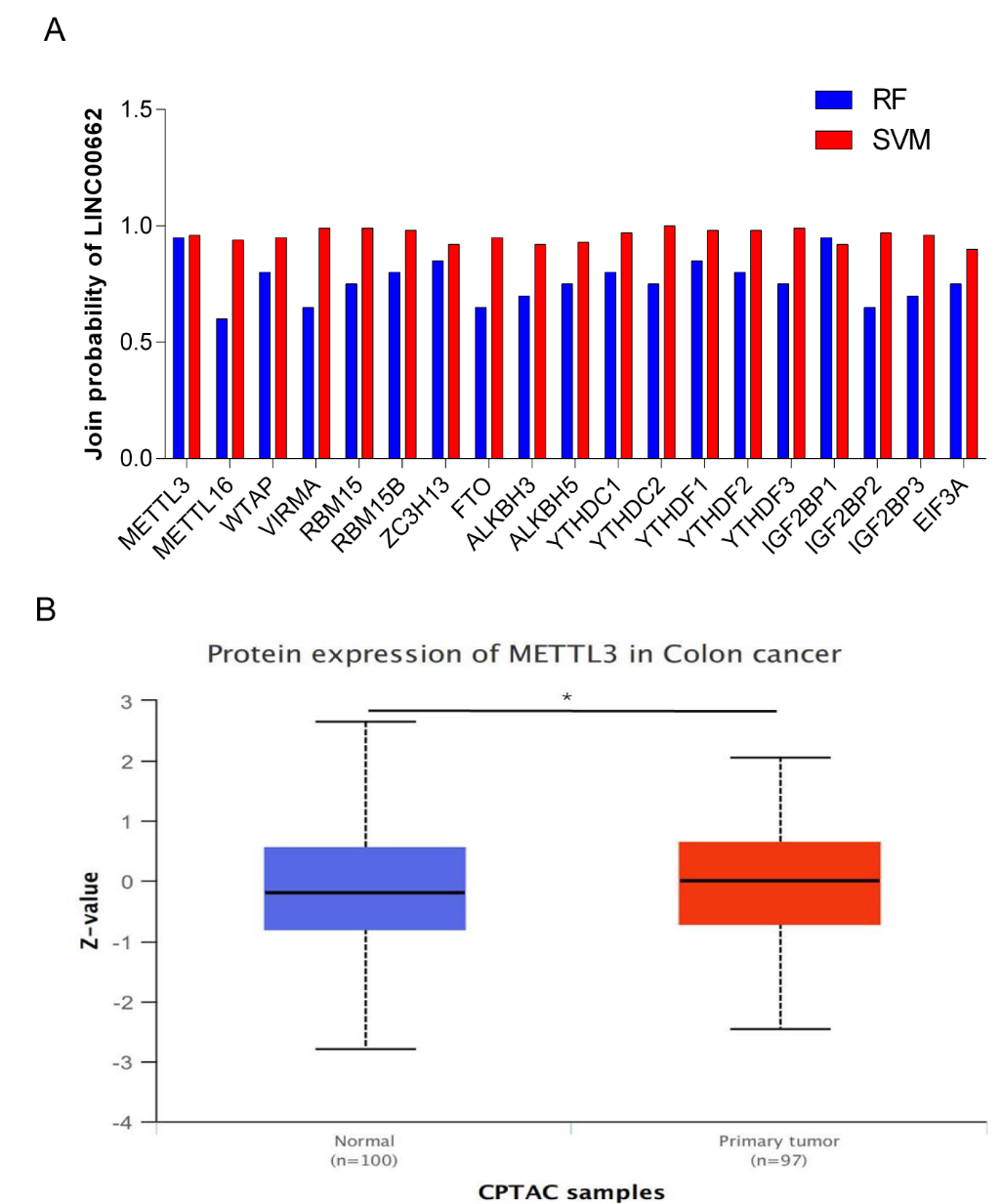
**Supplementary Fig. 1** Analysis based on the TCGA CRC database: METTL3 was significantly positively correlated with LINC00662 and VEGFA. **(A)** In RNA and protein interaction database (http://pridb.gdcb.iastate.edu/RPISeq/), the possibility of m6A enzyme interaction with LINC00662 was predicted, such as METTL3, METTL16, WTAP, VIRMA, RMB 15, RMB 15 b, ZC3H13, FTO, ALKBH3, AL KBH5, YTHDC1 YTHDC2, YTHDF1 YTHDF2, YTHDF3, IGF2BP1, IGF2BP2, IGF2BP3, EIF3A. **(B)** In the CPTAC protein database (http://ualcan.path.uab.edu/analysis-prot.html), the protein expression level of METTL3 was analyzed.


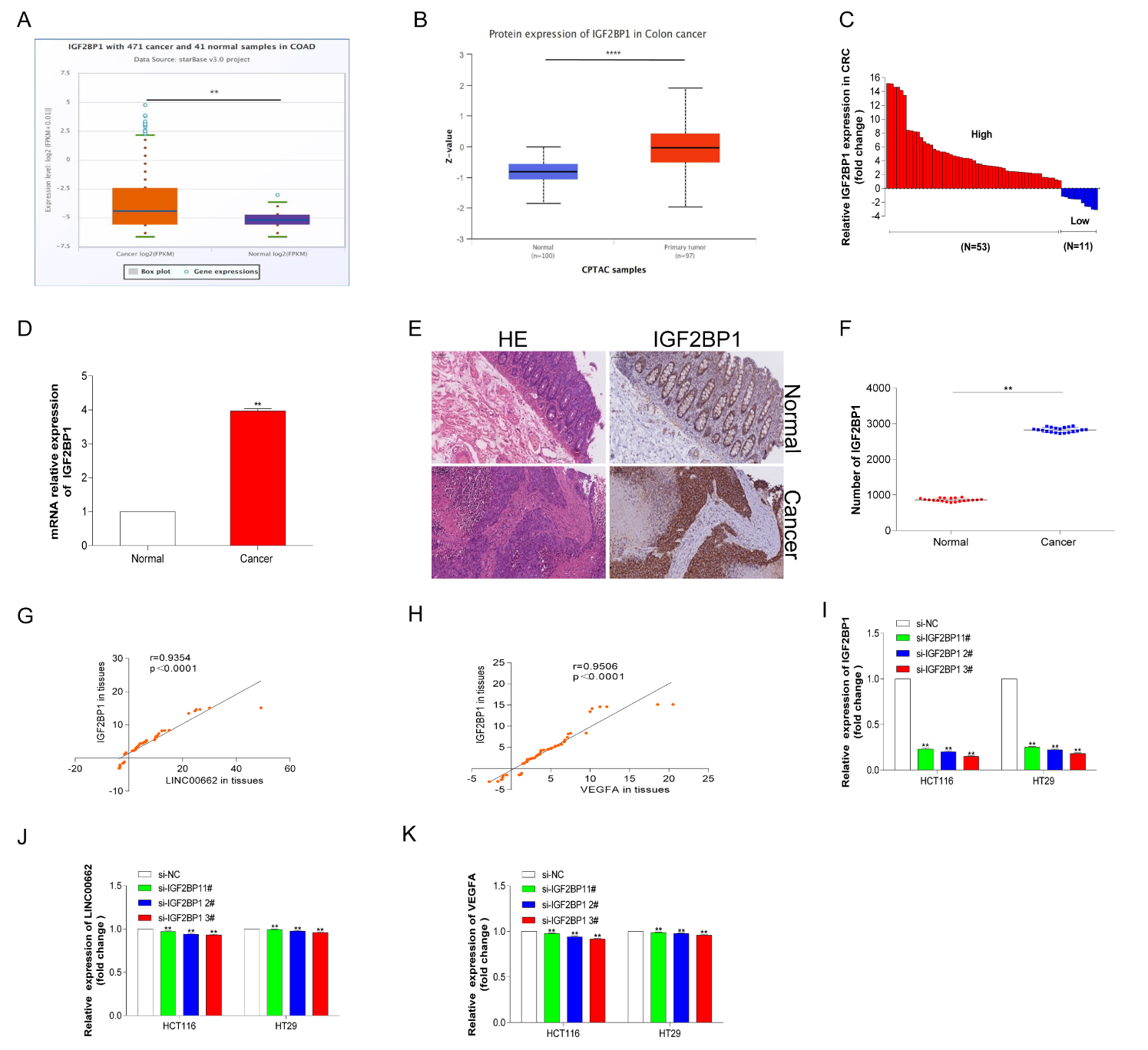
**Supplementary Fig. 2** METTL3 dual regulates LINC00662 and VEGFA RNAs stability. **(A)** In the TCGA starBase database for CRC, IGF2BP1 mRNA in CRC increased. **(B)** The expression levels were analyzed in the CPTAC IGF2BP1 protein database (<http://ualcan.path.uab.edu/analysis-prot.html>). **(C)** 64 pairs of CRC and adjacent tissues were selected. RNAs was extracted. The qRT-PCR results showed that the level of IGF2BP1 mRNA was highly expressed in CRC compared with adjacent tissues. **(D)** The average level of IGF2BP1 mRNA was highly expressed in CRC compared with adjacent tissues. **(E)** IHC staining and panoramic scanning for IGF2BP1 in CRC and para-cancer tissues **(F)** IHC analysis of 20 pairs of CRC and adjacent cancer showed that the m6A-related marker IGF2BP1 was significantly higher than that of adjacent cancer. **(G)** IGF2BP1 mRNA was significantly positively correlated with LINC00662. **(H)** IGF2BP1 mRNA was significantly positively correlated with VEGFA. **(I)** The expression level of IGF2BP1 was analyzed after interference with IGF2BP1 in HCT116 and HT29 cells. **(J)** The expression level of LINC00662 was analyzed after interference with IGF2BP1 in HCT116 and HT29 cells. **(K)** The expression level of VEGFA was analyzed after interference with IGF2BP1 in HCT116 and HT29 cells.

**Supplementary Table 1** The si-RNA sequence used in this study

| ID | Sequence (5'-3') |
| --- | --- |
| Si-LINC00662 1# | Sense: GCAUCCAUGAGGUGAUCAATT  Antisense: UUGAUCACCUCAUGGAUGCTT |
| Si-LINC00662 2# | Sense: CCAGCACCAAUUGUUAUAATT  Antisense: UUAUAACAAUUGGUGCUGGTT |
| Si-LINC00662 3# | Sense: GCUGCUGCCACUGUAAUAATT  Antisense: UUAUUACAGUGGCAGCAGCTT |
| Si-METTL3 1# | Sense: GGUUGCACGGUUCAAGCAA  Antisense: UUGCUUGAACCGUGCAACC |
| Si-METTL3 2# | Sense: CAGACGAAUUAUCAAUAAA  Antisense: UUUAUUGAUAAUUCGUCUG |
| Si-METTL3 3# | Sense: CAGUGGAUCUGUUGUGAUA  Antisense: UAUCACAACAGAUCCACUG |
| Si-IGF2BP1 1# | Sense: GGCUCAGUAUGGUACAGUA  Antisense: UACUGUACCAUACUGAGCC |
| Si-IGF2BP1 2# | Sense: AGCAAGAUACCGAGACAAA  Antisense: UUUGUCUCGGUAUCUUGCU |
| Si-IGF2BP1 3# | Sense: CGGUGAACGAGUUGCAGAA  Antisense: UUCUGCAACUCGUUCACCG |
